# Comparison of Four‐Dimensional Magnetic Resonance Imaging Analysis of Left Ventricular Fluid Dynamics and Energetics in Ischemic and Restrictive Cardiomyopathies

**DOI:** 10.1002/jmri.28076

**Published:** 2022-01-24

**Authors:** Alessandra Riva, Francesco Sturla, Silvia Pica, Antonia Camporeale, Lara Tondi, Simone Saitta, Alessandro Caimi, Daniel Giese, Giovanni Palladini, Paolo Milani, Serenella Castelvecchio, Lorenzo Menicanti, Alberto Redaelli, Massimo Lombardi, Emiliano Votta

**Affiliations:** ^1^ Department of Electronics, Information and Bioengineering Politecnico di Milano Milan Italy; ^2^ 3D and Computer Simulation Laboratory IRCCS Policlinico San Donato San Donato Milanese Italy; ^3^ Multimodality Cardiac Imaging IRCCS Policlinico San Donato San Donato Milanese Italy; ^4^ Siemens Healthcare GmbH Erlangen Germany; ^5^ Amyloidosis Research and Treatment Center, Fondazione IRCCS Policlinico San Matteo, Department of Molecular Medicine University of Pavia Pavia Italy; ^6^ Cardiac Surgery Department IRCCS Policlinico San Donato San Donato Milanese Italy

**Keywords:** 4D flow MRI, LV energetics, hemodynamic forces, light‐chain amyloidosis, ischemic cardiomyopathy

## Abstract

**Background:**

Time‐resolved three‐directional velocity‐encoded (4D flow) magnetic resonance imaging (MRI) enables the quantification of left ventricular (LV) intracavitary fluid dynamics and energetics, providing mechanistic insight into LV dysfunctions. Before becoming a support to diagnosis and patient stratification, this analysis should prove capable of discriminating between clearly different LV derangements.

**Purpose:**

To investigate the potential of 4D flow in identifying fluid dynamic and energetics derangements in ischemic and restrictive LV cardiomyopathies.

**Study Type:**

Prospective observational study.

**Population:**

Ten patients with post‐ischemic cardiomyopathy (ICM), 10 patients with cardiac light‐chain cardiac amyloidosis (AL‐CA), and 10 healthy controls were included.

**Field Strength/Sequence:**

1.5 T/balanced steady‐state free precession cine and 4D flow sequences.

**Assessment:**

Flow was divided into four components: direct flow (DF), retained inflow, delayed ejection flow, and residual volume (RV). Demographics, LV morphology, flow components, global and regional energetics (volume‐normalized kinetic energy [KE_V_] and viscous energy loss [EL_V_]), and pressure‐derived hemodynamic force (HDF) were compared between the three groups.

**Statistical Tests:**

Intergroup differences in flow components were tested by one‐way analysis of variance (ANOVA); differences in energetic variables and peak HDF were tested by two‐way ANOVA. A *P*‐value of <0.05 was considered significant.

**Results:**

ICM patients exhibited the following statistically significant alterations vs. controls: reduced KE_V_, mostly in the basal region, in systole (−44%) and in diastole (−37%); altered flow components, with reduced DF (−33%) and increased RV (+26%); and reduced basal–apical HDF component on average by 63% at peak systole. AL‐CA patients exhibited the following alterations vs. controls: significantly reduced KE_V_ at the E‐wave peak in the basal segment (−34%); albeit nonstatistically significant, increased peaks and altered time‐course of the HDF basal–apical component in diastole and slightly reduced HDF components in systole.

**Data Conclusion:**

The analysis of multiple 4D flow‐derived parameters highlighted fluid dynamic alterations associated with systolic and diastolic dysfunctions in ICM and AL‐CA patients, respectively.

**Level of Evidence:**

2

**Technical Efficacy Stage:**

3

Cardiac magnetic resonance imaging (MRI) is the reference standard imaging technique for morphofunctional assessment and noninvasive tissue characterization of cardiac diseases.^1^ It offers the possibility to acquire multiple sequences, providing insight into different and complementary aspects of left ventricular (LV) function: morphology and global/regional kinesis by cine sequences, tissue characterization by late gadolinium enhancement (LGE), T1 and T2 mapping,^2^ intracavitary blood fluid dynamics via 2D phase‐contrast MRI,^3^ and, more recently, via time‐resolved phase‐contrast MRI with three‐directional velocity encoding (4D flow).^4^ With suitable post‐processing, the latter potentially allows for the comprehensive quantification of LV fluid dynamics, including those parameters related to the inherent 3D nature of LV blood flow: flow components subdivision,^5^, ^6^ energy distribution,^7^, ^8^ pressure gradients, and hemodynamic forces (HDFs).^9^

4D flow is increasingly employed in clinical practice and research^10^: multiple studies suggest that LV morphology, tissue composition, wall contractility, and compliance are tightly linked to intracavitary fluid dynamics.^6^, ^11^, ^12^, ^13^, ^14^ Thus, the quantification of fluid dynamic derangements associated with LV myocardial diseases can improve the mechanistic understanding and interpretation of disease progression.

According to the etiology of LV cardiomyopathy, the pattern of chamber remodeling may differ. For instance, ischemic cardiomyopathy (ICM), secondary to coronary artery disease and myocardial infarction, is the most common type of dilated cardiomyopathy and is characterized by myocardial scarring, progressive chamber dilation, and impaired systolic function.^15^, ^16^ Differently, light‐chain cardiac amyloidosis (AL‐CA) is a progressive restrictive cardiomyopathy and the most serious form of amyloid involvement of the heart, marked by extracellular deposition of monoclonal kappa or lambda light chains. AL‐CA is a predominant diastolic dysfunction associated with wall thickening, slowed LV relaxation, increased myocardial stiffness, and reduced compliance.^17^, ^18^

Given the expected interplay between LV wall function and intracavitary flow, we hypothesized that different LV pathological conditions, such as ICM and AL‐CA, may exhibit a different pattern of intracavitary flow derangements.

Previous studies have already employed 4D flow to assess hemodynamic derangements associated with pathological LV conditions. Garg *et al*. showed that post‐ischemic patients have reduced global LV kinetic energy (KE),^8^ and LV impairment strongly associated with the reduction of systolic KE,^13^ together with a delay in the systolic KE peak, is also present in post‐ischemic patients with preserved ejection fraction (EF). For ICM patients, Stoll *et al*.^19^ reported a remarkable decrease of the direct‐flow component (DF, blood that enters and exits the LV in the same cardiac cycle) compared to controls, and this decrease in DF corresponded to a similar increase in the residual volume (RV, blood that remains in the LV for at least two cardiac cycles) component compared to controls. According to Arvidsson and coworkers,^20^ patients with heart failure and LV mechanical dyssynchrony exhibited significantly altered HDF patterns compared to normal subjects. In a heterogeneous cohort of heart failure patients, Eriksson *et al*.^21^ identified specific HDF derangements associated with left bundle branch block conduction abnormality.

However, although previous studies demonstrated the relevance of specific 4D flow parameters, no study has investigated LV flow components,^5^ energetics,^8^, ^22^ and pressure‐driven HDFs^9^ as complementary and interdependent aspects of LV fluid dynamics. Furthermore, to date, LV fluid dynamic derangements associated with AL‐CA remain unexplored through 4D flow.

Hence, the aims of this study were to: 1) investigate the potential of 4D flow to identify derangements in fluid dynamic features specifically associated with ICM and AL‐CA as examples of depressed LV systolic function and altered LV diastolic compliance, respectively, and 2) evaluate if the combined analysis of multiple fluid dynamic features provides extra insight into the underlying pathophysiological mechanisms.

## Materials and Methods

### Study Population

The study conformed to the Ethical Guidelines of the Declaration of Helsinki; all subjects provided written informed consent.

Ten ICM patients, 10 AL‐CA patients, and 10 controls with no history of cardiac disease and no cardiovascular risk factors were included in the study.

ICM was defined based on the history of previous anterior myocardial infarction, LV dilation, and systolic dysfunction (LV ejection fraction, i.e., EF below 45%) assessed by 2D echocardiography and MRI; severe aortic or mitral valvulopathies were excluded.

Cardiac involvement in AL‐CA patients was defined according to the established consensus criteria.^23^ All patients had a biopsy‐proven diagnosis of systemic amyloidosis and amyloid deposits were typed with immune‐electron microscopy or proteomics. A complete clinical, echocardiographic, and laboratory assessment was performed. Also, 2D echocardiography was performed following standard protocols; cardiac involvement was defined in case of increased LV wall thickness (>12 mm) or normal LV thickness with diastolic dysfunction and raised serum biomarkers.^23^

Blood samples for hematocrit were obtained at the time of MRI.

### MRI Acquisitions

All subjects underwent MRI at 1.5 T using a MAGNETOM Aera (Siemens Healthcare, Erlangen, Germany) scanner. A balanced steady‐state free precession (b‐SSFP) cine sequence with retrospective ECG gating was used to acquire short‐axis (SAx) and long‐axis (LAx, i.e., horizontal long‐axis [HLA], vertical long‐axis [VLA], and LV outflow tract [LVOT] views) LV views with the following parameters: TE (Echo Time) = 1.1–1.8 msec, TR (Repetition Time) = 2.0–5.9 msec (segments = 14–16), slice thickness = 8 mm (no gap), pixel spacing = 1.25–1.65 mm, and reconstructed temporal resolution = 22–46 msec (reconstructed phases = 30). ICM and AL‐CA patients were administered with a bolus of gadobutrol (0.1 mmol/kg, Gadovist, Bayer Schering Pharma, Berlin, Germany). No contrast agent was used in controls.

In the same session, 4D flow images were acquired using a prototype time‐resolved 3D gradient echo sequence with three‐directional velocity encoding a parasagittal‐oriented field of view covering the entire LV. 4D flow was performed during free‐breathing with retrospective ECG‐triggering and adaptive respiratory gating. The following scan parameters were implemented: velocity encoding = 80–150 cm/sec to limit aliasing, flip angle = 7°(without contrast) to 15°(with contrast), TE = 2.3–2.8 msec, TR = 4.8–5.2 msec (segments = 2), voxel size = (2.0 × 2.0 × 2.0) to (2.8 × 2.8 × 2.8) mm^3^, reconstructed temporal resolution = 22–65 msec, and reconstructed phases = 20 (9 subjects) or 30 (21 subjects). 4D flow data were corrected for eddy currents and aliasing. All patients were in sinus rhythm during MR acquisition.

### Image Analysis

**FIGURE 1:**
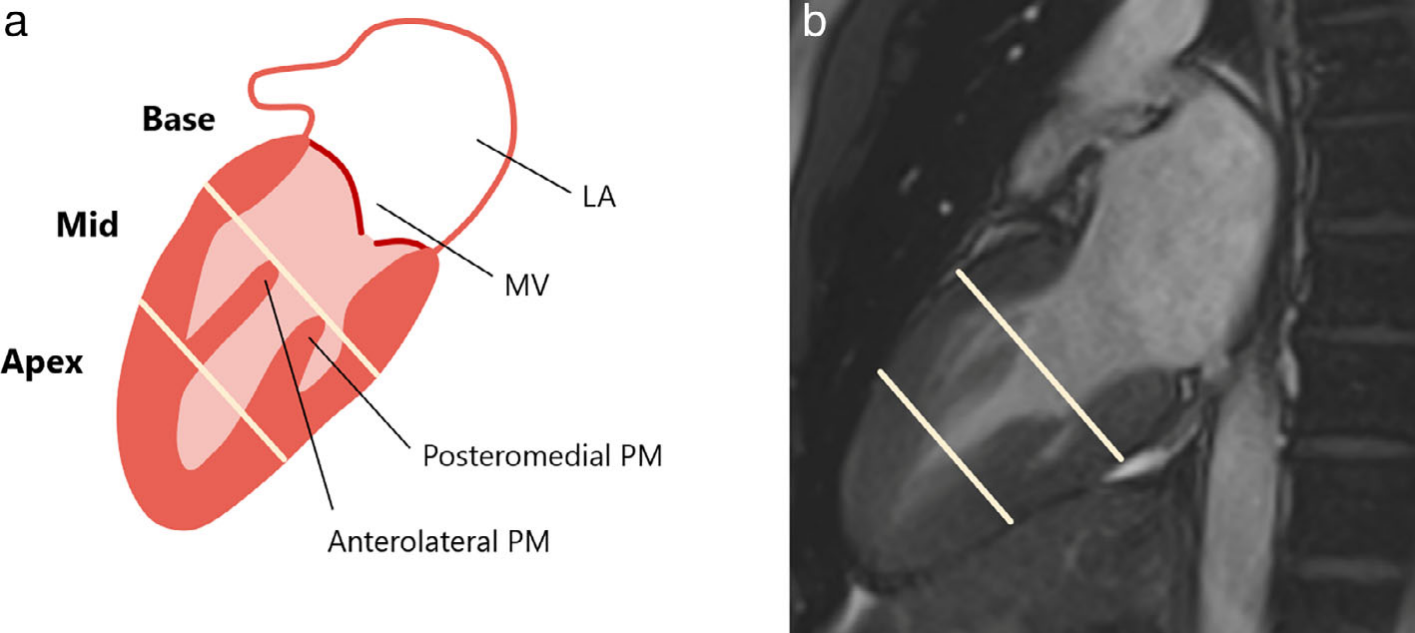
**(a)** Illustration of vertical long‐axis (VLA, two‐chamber) LV view with anatomic landmarks for the selection of basal (mitral valve to papillary muscle [PM] tips), mid (PM tips to PM base), and apical (below PM base) regions; **(b)** MR appearance of PMs from VLA view and subsequent regional subdivision.

LV volumes, mass, and EF were calculated from b‐SSFP cine images using a thresholding method in Medis (Qmass MR v6.2.1, Medis, Leiden, The Netherlands) by an expert cardiologist (S.P., 15 years of experience). Also, an expert operator (A.R., 4 years of experience) employed b‐SSFP images to reconstruct dynamic LV masks as in a previous work^24^; further details are available in Appendix S1 in the Supplemental Material. In LAx views, the tip and base of each papillary muscle (PM; Fig. 1a) and mitral and aortic annular points were traced at end diastole and automatically tracked over the cardiac cycle (Fig. S1 in the Supplemental Material) on LAx views, i.e., HLA, VLA, and LVOT views. PMs were used to define LV basal, mid‐cavity, and apical regions for regional analysis (Fig. 1b).^25^

### LV Flow Analysis

For each condition, LV flow subdivision, LV energetics in terms of KE and viscous energy loss (EL), and LV HDF were quantified (Table 1); A.R. oversaw the entire process of computation.

**TABLE 1:** Summary of the Computed Fluid Dynamic Parameters

Parameters		Definition	Implication on Pathophysiology
Flow subdivision (%)		Distribution of separate flow transit components^5^: Direct flow Retained inflow Delayed ejection flow Residual volume	Alteration of the repartition of flow components may prove useful as subclinical marker of impaired chamber function^6^
Energetics	KE (J)	Kinetic energy associated with blood motion^8^	Altered KE indicates an altered intracavitary blood mean velocity, hence an alteration in the capability of LV myocardium to accelerate/decelerate blood. Yet, KE does not reflect the direction/organization of blood motion
	EL (J)	Part of KE dissipated due to blood viscosity (frictional viscous forces) and the presence of velocity gradients in bulk of the intracavitary blood flow^22^	Alteration of EL may be related to abnormal vortical structures or less energy‐efficient blood transportation
Hemodynamic force (N)		Resultant load exerted by the intracavitary blood on the myocardial wall due to pressure gradients.^30^ The opposite of this vector (action–reaction law) reflects the net transfer of momentum from the LV wall to the intracavitary blood	Derangements in force direction or magnitude may reflect the imbalance or the lack of synchronicity of the contraction of different regions of the myocardial wall (in systole), or of their compliance (in diastole)

#### FLOW COMPONENTS

The intraventricular blood flow was automatically divided into four components using cvi^42^ (Circle Cardiovascular Imaging, Calgary, Canada) according to Eriksson *et al*.^26^ (Fig. S2 in the Supplemental Material): 1) DF, blood that enters the LV during diastole and leaves during systole in the same heartbeat; 2) retained inflow (RI), blood entering the LV but not leaving in the analyzed heartbeat; 3) delayed ejection (DE) flow, blood already in LV during diastole that leaves during systole; 4) RV, blood that resides within the LV for at least two cardiac cycles. The volume of the flow components was expressed as a fraction of LV end‐diastolic volume.

#### INTRAVENTRICULAR ENERGETICS

KE and EL (the portion of KE irreversibly lost due to frictional viscous forces) were computed for each LV region and for the whole chamber.^22^ At each time frame, blood KE, i.e., the energy that a volume of blood possesses due to its motion, was computed as the sum of $\frac{1}{2}mv^{2}$ for each voxel, where *m* is the mass of blood in one voxel and *v* is the velocity magnitude in each voxel.^27^

#### HEMODYNAMIC FORCE

The pressure gradient (***b***) was computed from the Navier–Stokes equation.^28^ The HDF vector was calculated for every time frame by integrating ***b*** over the entire LV volume and was then projected onto three mutually orthogonal directions (Fig. S3 in the Supplemental Material): 1) basal–apical (HDF_basal–apical_), perpendicular to the atrioventricular plane and defined from apex and mitral landmarks on the HLA view; 2) septal–lateral (HDF_septal–lateral_), parallel to the LVOT view; and 3) inferior–anterior (HDF_inferior–anterior_), perpendicular to the septal–lateral and basal–apical directions.^29^ The root mean square (RMS) values of HDF_septal–lateral_, HDF_inferior–anterior_, and HDF_basal–apical_ were computed over the cardiac cycle. The three values were combined to derive the ratio between the RMS of the transversal and basal–apical components of the HDF (*R*_RMS_)^29^ (further details are available in Appendix S1 in the Supplemental Material).

For all calculations, blood was assumed to be a non‐Newtonian fluid with density equal to 1025 kg/m^3^ and viscosity μ dependent on patient‐specific hematocrit and shear rate, i.e., the velocity gradient between adjacent layers of blood^24^ (further details are available in Appendix S1 in the Supplemental Material).

At each time‐point, KE, EL, and HDF were normalized to the current LV volume to account for the potentially confounding effect of heart size.^30^ Region‐specific (i.e., basal, mid, and apical) KE and EL values were normalized to the current volume of the corresponding region.

### Analysis of Interoperator Variability

Our analyses are characterized by two manual steps of the processing pipeline: 1) the manual initialization of the semiautomatic segmentation of the LV endocardium on cine MR images, and, when needed, 2) the manual corrections of the LV endocardial contour yielded by the automated tracking algorithm. These steps can impact the binary masks defined in the 4D flow data to identify the LV chamber. We assessed their combined impact via interobserver variability analysis of the binary masks obtained upon segmentation by two independent and double‐blinded operators (A.R. and F.S., 4 and 9 years of experience, respectively) of the data of 15 randomly selected subjects. Also, peak values of KE_V_ and HDF, computed based on the different binary masks, were statistically compared. Further details are reported in Appendix S1 in the Supplemental Material.

### Statistical Analysis

Demographic and standard MRI measures are reported as mean ± SD. Normality of the distribution of continuous data was assessed through the Shapiro–Wilk test. One‐way analysis of variance (ANOVA) with Tukey's post hoc test was used to compare the three groups in terms of flow components. Changes in energetic variables and in HDF peak values were assessed using two‐way ANOVA.

In the interoperator analysis, the Dice Score Coefficient (DSC) was computed to compare the LV binary masks obtained by the two independent operators; also, linear regression was adopted for KE and HDF components computed based on different LV masks.

Statistical analyses were performed with GraphPad Prism 8 (GraphPad Software Inc., La Jolla, CA, USA); a *P*‐value of <0.05 was considered significant.

## Results

Characteristics of the three groups are detailed in Table 2; no statistically significant difference in age was noticed (*P* = 0.055; 95% CI: 54–63 in controls, 59–70 in ICM and 61–72 in AL‐CA). ICM patients had significantly greater LV volumes and lower EF with respect to both controls and AL‐CA patients; AL‐CA patients had significantly lower stroke volume (SV) compared to control subjects.

**TABLE 2:** Complete Study Population Characteristics

	Controls (*N* = 8)	ICM (*N* = 10)	AL‐CA (*N* = 10)	*P*‐value	Tukey *P*‐value		
					C vs. ICM	C vs. AL‐CA	ICM vs. AL‐CA
Age (years)	58 ± 7	64 ± 8	67 ± 8	0.055	0.192	0.052	0.784
BSA (m^2^)	1.9 ± 0.2	2.0 ± 0.2*	1.7 ± 0.2	0.009	0.329	0.160	0.007
Sex (F, %)	3 (30)	1 (10)	6 (67)	0.058	—	—	—
Heart rate (bpm)	67 ± 12	66 ± 13*	80 ± 10	0.036	0.973	0.079	0.048
LV EDV_i_ (mL/m^2^)	67 ± 12**	124 ± 34*	54 ± 12	<0.0001	<0.0001	0.385	<0.0001
LV ESV_i_ (mL/m^2^)	21 ± 5**	86 ± 35*	20 ± 9	<0.0001	<0.0001	0.991	<0.0001
LV SV_i_ (mL/m^2^)	46 ± 8	38 ± 10	34 ± 9	0.019	0.133	0.016	0.588
LV EF (%)	69 ± 5**	32 ± 10*	64 ± 13	<0.0001	<0.0001	0.455	<0.0001
CI (L/min/m^2^)	3.0 ± 0.6	2.5 ± 0.7	2.7 ± 0.7	0.207	0.183	0.542	0.735
LV mass_i_ (g/m^2^)	61.9 ± 9.8* ^,^ **	94.5 ± 19.9	96.3 ± 30.6	0.002	0.007	0.004	0.981
Hematocrit (%)	43 ± 3	40 ± 6	39 ± 5	0.114	0.295	0.109	0.836

Data are expressed as mean ± SD.

AL‐CA = light‐chain cardiac amyloidosis; BSA = body surface area; bpm = beats per minute; C = controls; ICM = ischemic cardiomyopathy; LV = left ventricle; EDV = end‐diastolic volume; ESV = end‐systolic volume; SV = stroke volume; EF = ejection fraction; CI = cardiac index.

Two‐way ANOVA for repeated measures (Tukey multiple comparisons test):

** *P* < 0.05 vs. ICM;

* *P* < 0.05 vs. AL‐CA.

### Flow Components

**FIGURE 2:**
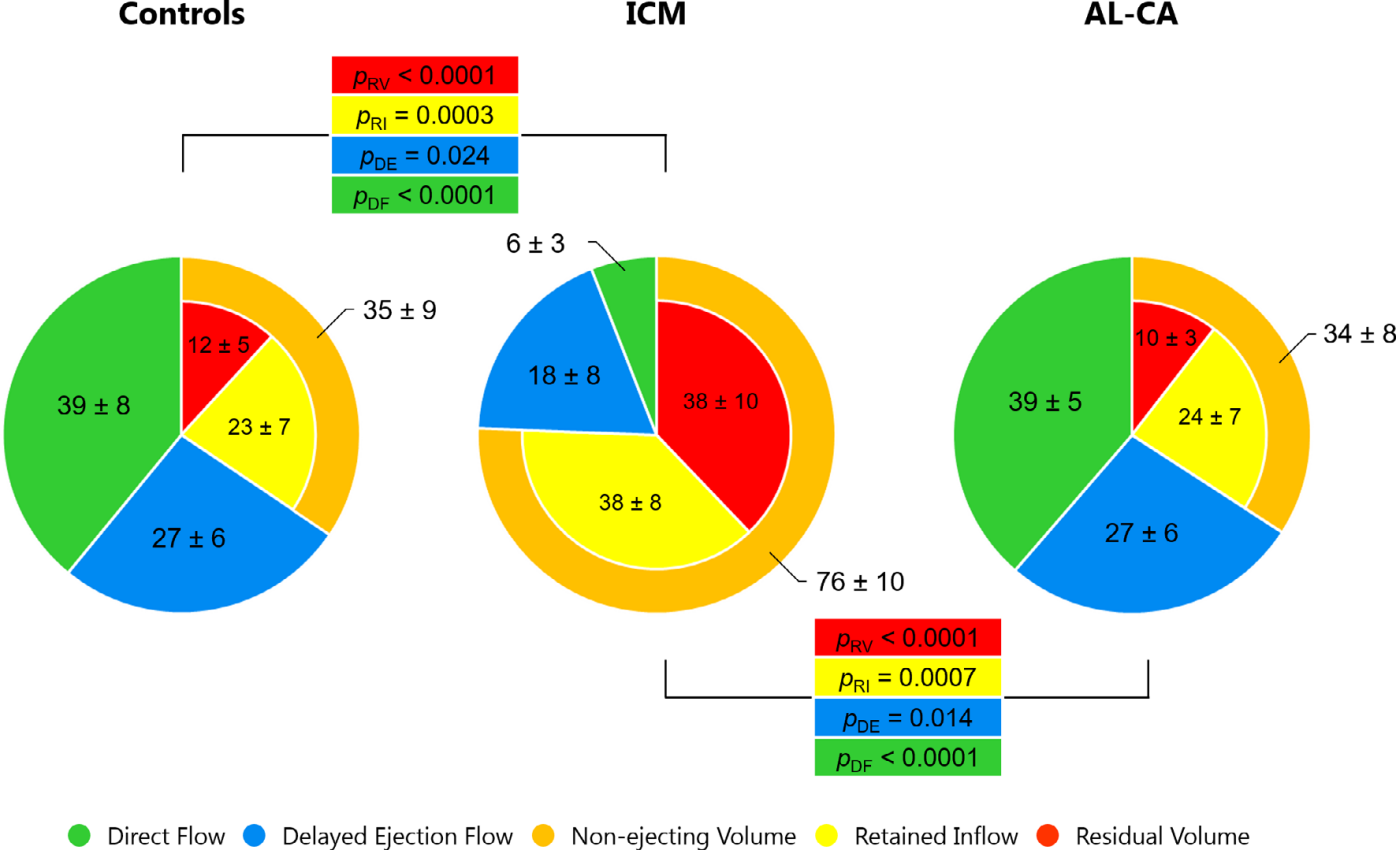
LV flow components as percentage of LV end‐diastolic volume (expressed as mean ± SD) for the study population. The significant Tukey post hoc *P*‐values are reported for controls vs. ICM and ICM vs. AL‐CA. AL‐CA, light‐chain cardiac amyloidosis; DE, delayed ejection flow; DF, direct flow; ICM, ischemic cardiomyopathy; RI, retained inflow; RV, residual volume.

**FIGURE 3:**
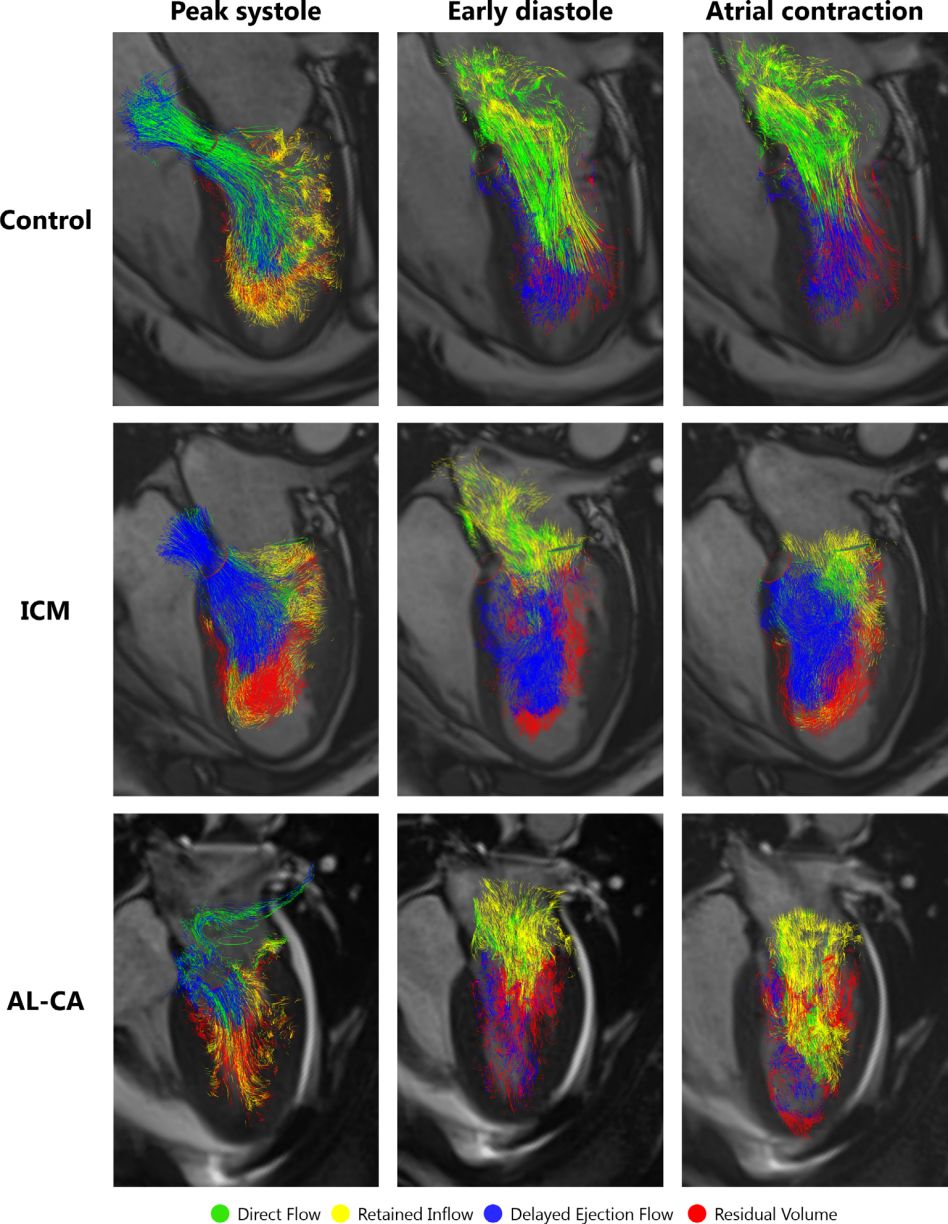
Blood flow pathline visualization of the four flow components throughout the cardiac cycle for a healthy 60‐year‐old man, a 65‐year‐old male with ICM and a 64‐year‐old woman with AL‐CA. Semitransparent HLA images provide anatomical orientation. AL‐CA, light‐chain cardiac amyloidosis; HLA, horizontal long‐axis; ICM, ischemic cardiomyopathy.

Compared to controls, the overall partition in flow components in AL‐CA patients was preserved, while ICM subjects showed significantly reduced DF and DE and significantly increased RI and RV compared to both AL‐CA subjects and controls (Figs 2 and 3, Video S1 in the Supplemental Material).

### LV Energetics

#### TIME‐DEPENDENT KE_V_

**FIGURE 4:**
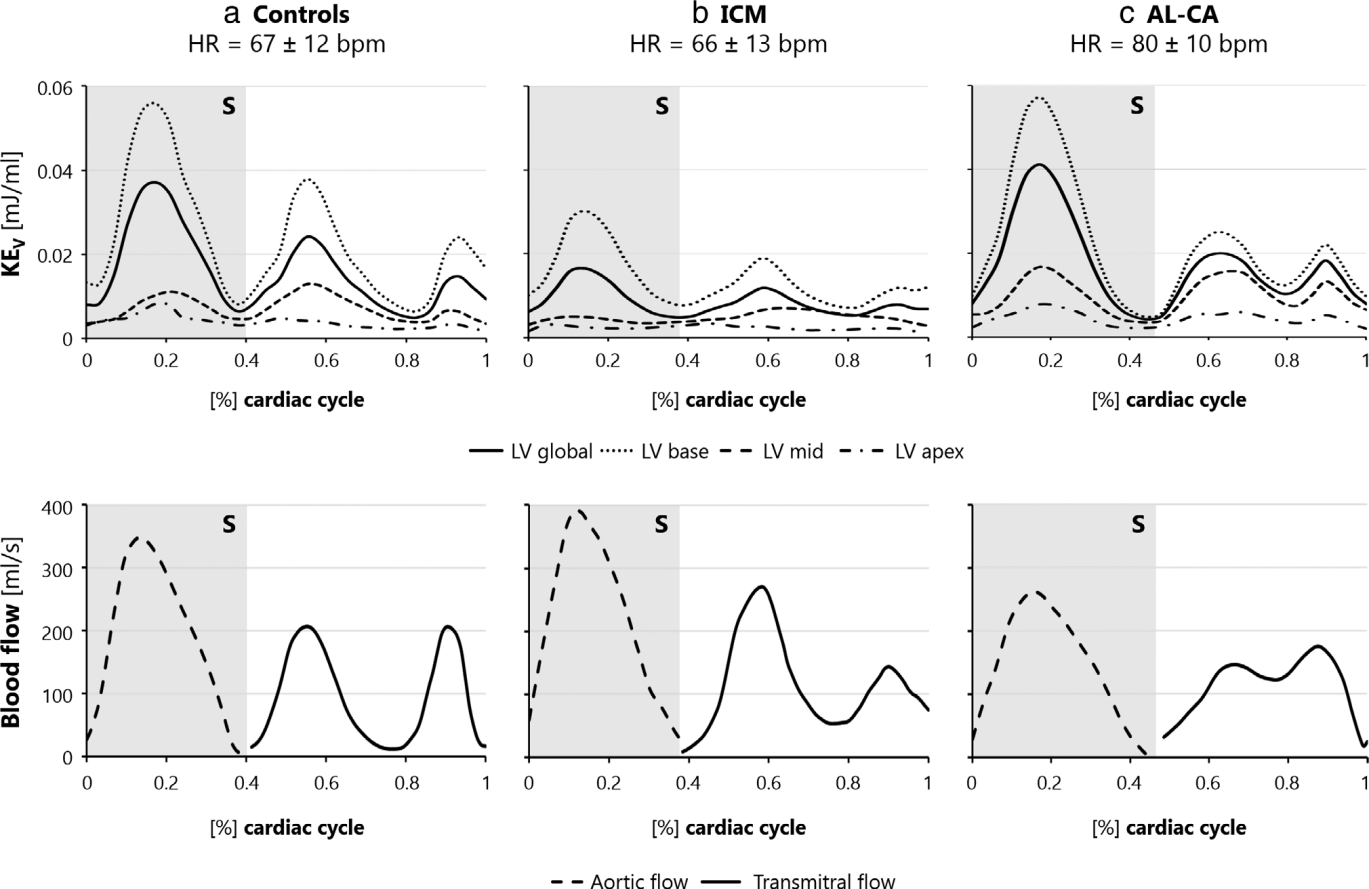
Top panel: Group‐averaged KE_V_ during the cardiac cycle, starting at end‐diastole for controls **(a)**, ICM **(b)**, and AL‐CA **(c)** for the entire LV and basal, mid‐cavity, and apical regions. Bottom panel: Aortic flow curves (dashed line) from end‐diastole to end‐systole, and transmitral flow curves (solid line), starting at end‐systole. AL‐CA, light‐chain cardiac amyloidosis; bpm, beats per minute; HR, heart rate; ICM, ischemic cardiomyopathy; KE_V_, kinetic energy normalized to volume; LV, left ventricle; S, systole.

In controls, the time‐course of KE_V_ showed a peak during systole, a peak during the early rapid filling phase of diastole (E‐wave), and a subsequent small but clearly separated peak, corresponding to atrial contraction (A‐wave) (Fig. 4a). In ICM patients, the global systolic peak was significantly reduced (Table S1 in the Supplemental Material), and the physiological two‐peak diastolic pattern was lost (Fig. 4b). AL‐CA patients showed a comparable global systolic peak vs. controls (*P* = 0.92; Fig. 4c, Table S1 in the Supplemental Material); however, a significant reduction in the amplitude of the diastolic E‐wave peak was observed (Table S1 in the Supplemental Material).

The mid and basal regions were the principal contributors to global KE_V_ (Fig. 4, top panel). In ICM patients, both local contributions were significantly reduced vs. controls in terms of systolic and E‐wave peaks. In AL‐CA patients, a reduction (*P* = 0.04) in KE_V_ was noticed in the basal region for E‐wave peak (Table S1 in the Supplemental Material).

#### TIME‐AVERAGED KE_V_

**FIGURE 5:**
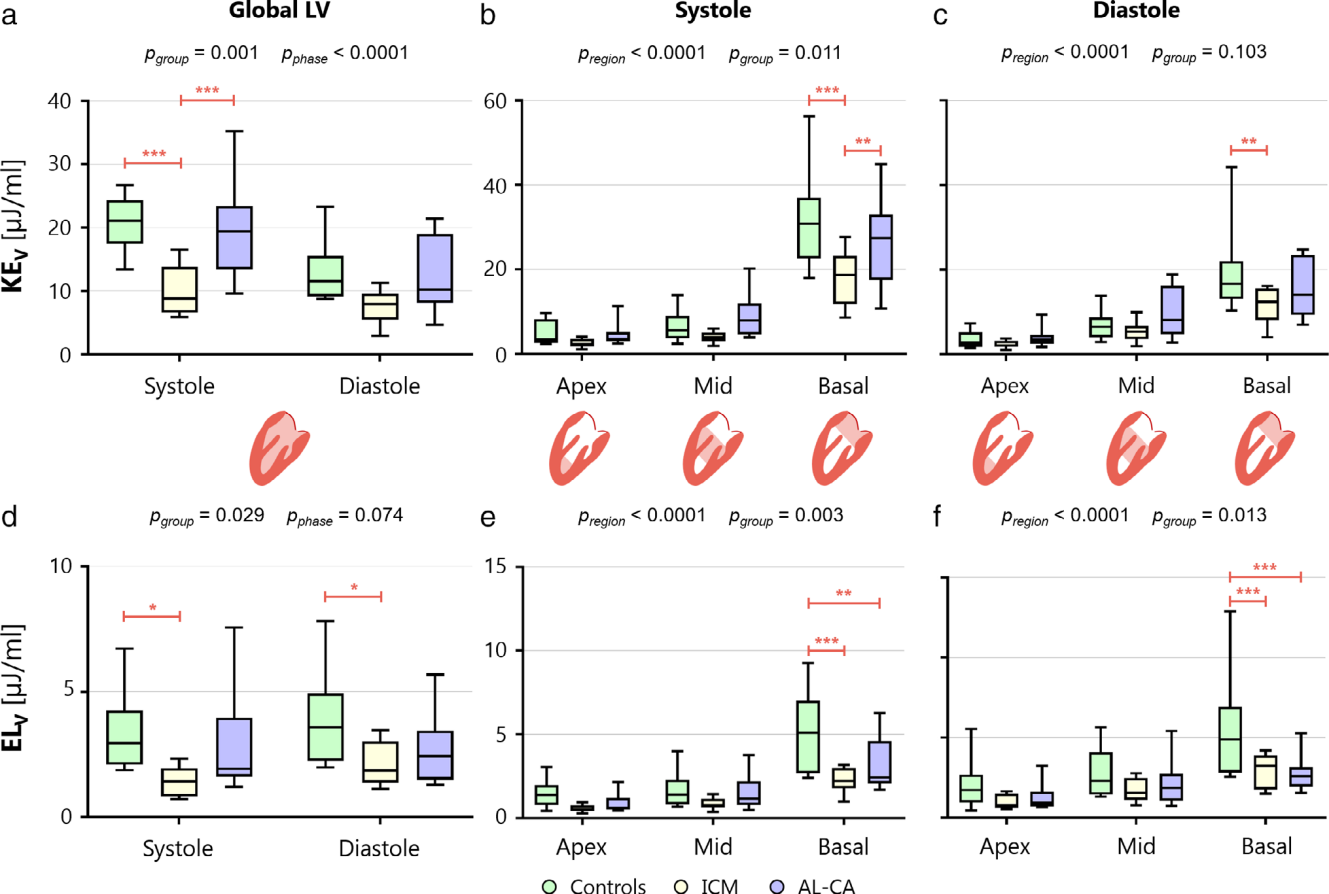
KE_V_ (top panel) and EL_V_ (bottom panel) for the entire LV and basal, mid‐cavity, and apical regions, represented through box and whiskers plots. Each box ranges between 25^th^ and 75^th^ percentile with a line pointing out the median value; whiskers indicate the 10^th^ and the 90^th^ percentile, respectively. **(a, d)** KE_V_ and EL_V_ distribution for global LV, over systole and diastole. **(b, e)** KE_V_ and EL_V_ distribution at regional scale, over systole. **(c, f)** KE_V_ and EL_V_ distribution at regional scale, over diastole. Solid black line represents the mean value for each population. AL‐CA, light‐chain cardiac amyloidosis; EL_V_, viscous energy loss normalized to volume; ICM, ischemic cardiomyopathy; KE_V_, kinetic energy normalized to volume; LV, left ventricle. *P < 0.05; **P < 0.01; ***P < 0.001.

Global KE_V_ was significantly different between systole and diastole, and there were significant intergroup differences (Fig. 5a). In particular, systolic KE_V_ was significantly decreased in both ICM and AL‐CA compared to controls.

In systole, significant inter‐region differences in KE_V_ were detected within each group as well as between groups (Fig. 5b). In particular, KE_V_ in the basal region was significantly reduced in ICM patients compared to controls and AL‐CA patients.

In diastole (Fig. 5c), there were significant inter‐region differences. Despite negligible intergroup differences (*P*_group_ = 0.103), KE_V_ associated with the basal region was significantly reduced in ICM vs. controls.

#### VISCOUS ENERGY LOSS (EL_V_)

In each group, global EL_V_ was comparable between systole and diastole (*P*_phase_ = 0.074). Yet, in both phases, significant intergroup differences were observed (Fig. 5d). Post hoc analysis highlighted that these were particularly evident between ICM patients and controls: in ICM patients, the median EL_V_ value was reduced by 52% and 48% in systole and diastole, respectively.

When focusing on regional EL_V_, significant inter‐region differences were observed in systole and in diastole; in both phases, EL_V_ increased when moving from the apex to the mid and to the basal regions. Also, significant intergroup differences were observed in both systole and diastole on a region‐specific basis (Fig. 5e,f). In both phases, EL_V_ in the basal zone was significantly lower with respect to controls in ICM and AL‐CA patients.

### Hemodynamic Force

**FIGURE 6:**
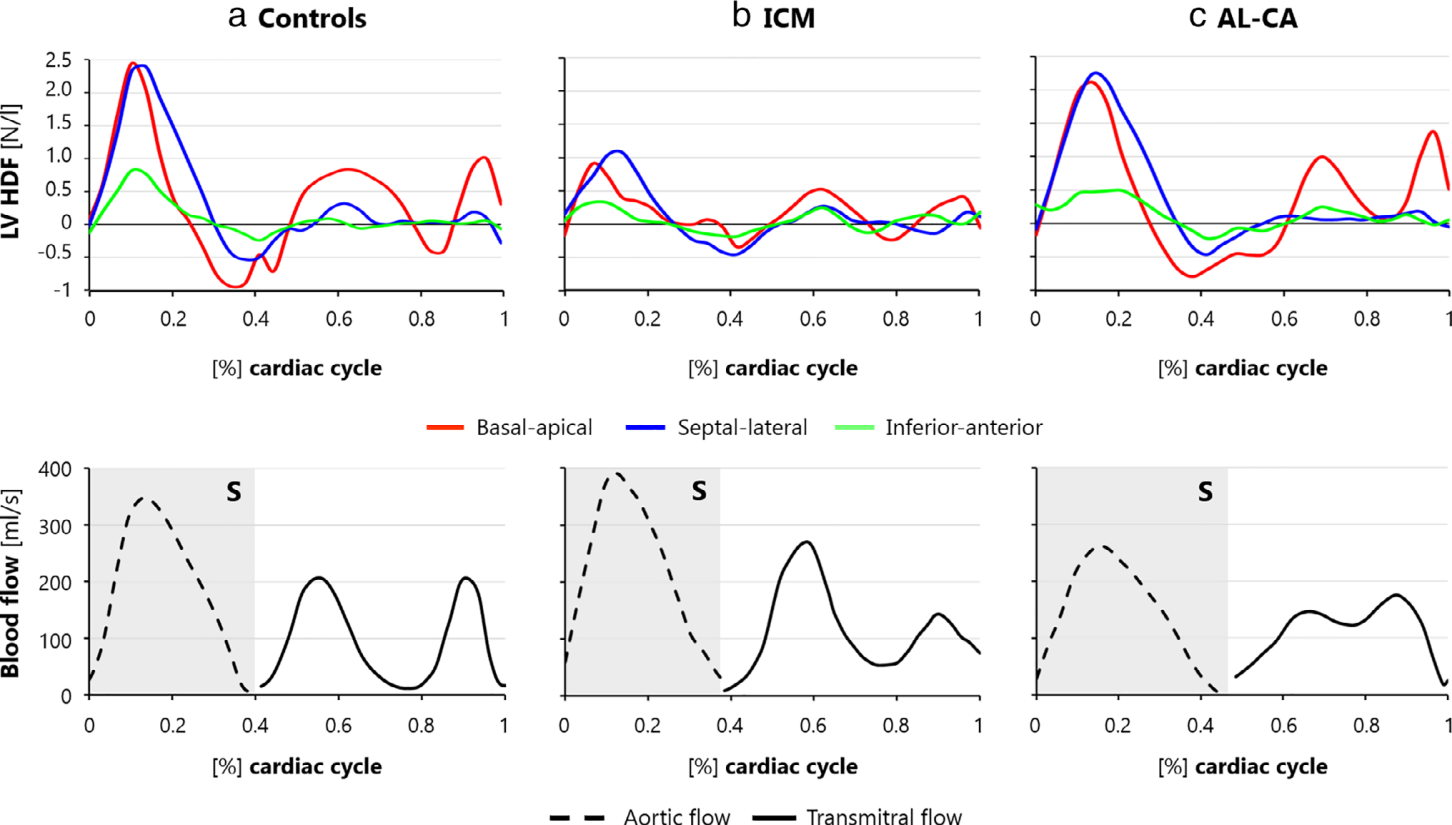
Top panel: Average HDF normalized to LV volume curves (N/L) for the three components: basal–apical (red; increasing values indicate a force oriented toward the apex), septal–lateral (blue; increasing values indicate a force oriented toward the lateral wall), and inferior–anterior (green; increasing values indicate a force oriented toward the anterior wall). Bottom panel: aortic flow curves (dashed line) from end‐diastole to end‐systole, and transmitral flow curves (solid line), starting at end‐systole. Curves are represented for controls **(a)**, ICM **(b)**, and AL‐CA **(c)**. AL‐CA, light‐chain cardiac amyloidosis; HDF, hemodynamic force; ICM, ischemic cardiomyopathy; S, systole.

**FIGURE 7:**
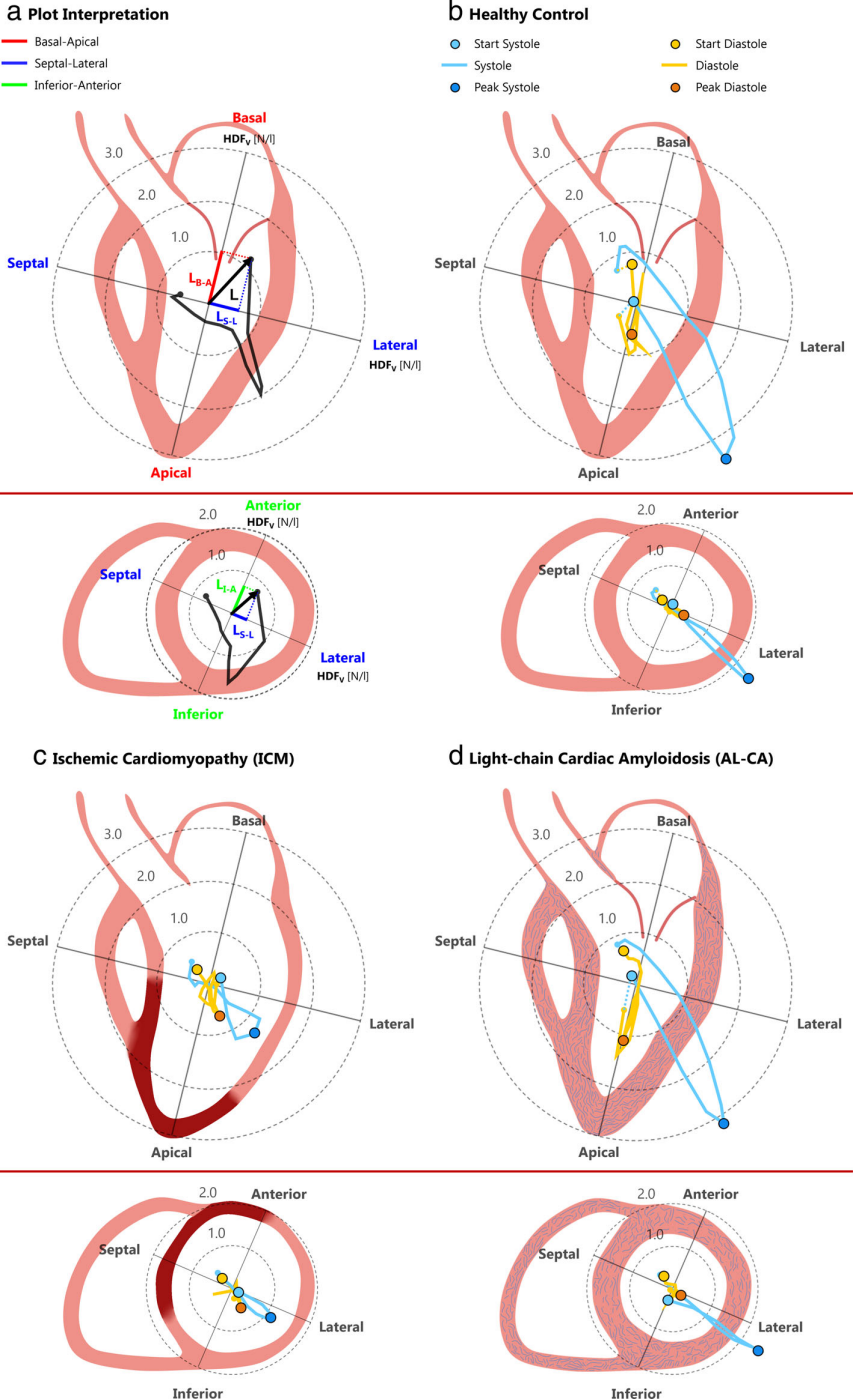
Magnitude and orientation of the volume‐normalized hemodynamic force (HDF) (N/L) over the cardiac cycle. **(a)** Plot interpretation: data are presented through polar plots in the LVOT LAx view (upper figure) and in the mid SAx view (lower figure) by projecting the HDF on these two mutually orthogonal views. In each view, the time‐dependent position of the tip of the relevant HDF projection over the cardiac cycle is reported; the angular position of the point and its distance from the origin represent the direction and the magnitude of the vector, respectively. The center of the polar plot not necessarily represents the real point of application of the HDF, which is by definition the center of mass of LV intracavitary blood, and hence moves over the cardiac cycle. **(b–d)** Group‐averaged plots for controls, ICM patients, and AL‐CA patients, respectively. The systolic phase is represented in light blue and the diastolic phase is represented in orange. For ICM, the red zone represents the myocardial scar, located in antero‐septal position. For AL‐CA, the light blue filaments represent the amyloid deposition between myocardial cells.

In controls, the time‐course of group‐averaged HDF components (Fig. 6a) showed a major and steep peak in HDF_basal–apical_ and a concomitant peak in HDF_septal–lateral_ in systole. Accordingly, HDF was tilted toward the LVOT (Fig. 7b) due to the comparable magnitude of the HDF_basal–apical_ and HDF_septal–lateral_ components. In diastole, E‐wave and A‐wave peaks were clearly visible in HDF_basal–apical_. Concomitantly, the magnitude of HDF_septal–lateral_ and HDF_inferior–anterior_ remained negligible, thus maintaining the HDF vector aligned with the LV long‐axis.

**FIGURE 8:**
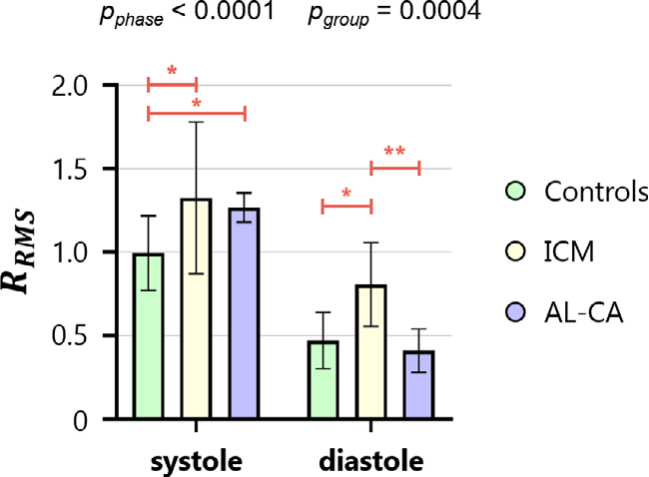
Bar chart of the *R*_RMS_ ratio in systole and diastole for each population. Bars represent mean ± SD. AL‐CA, light‐chain cardiac amyloidosis; ICM, ischemic cardiomyopathy; *R*_RMS_, root mean square ratio. *P < 0.05; **P < 0.01.

Qualitative assessment of the mean time‐course of group‐averaged HDF components suggested that in ICM patients the magnitude of each HDF component was smaller than in controls over the whole cardiac cycle (Fig. 6b). However, the intergroup statistical analysis of HDF components at specific time‐points provided a different indication (Table S2 in the Supplemental Material): at peak systole, a statistically meaningful reduction in each HDF component was observed (by 60% in HDF_septal–lateral_, 58% in HDF_basal–apical_, and 54% in HDF_inferior–anterior_). In diastole, instead, a statistically significant reduction vs. controls was observed only in E‐wave peak of HDF_basal–apical_; the reduction in the A‐wave peak of HDF_basal–apical_ was not statistically significant (*P* = 0.06). No statistically significant alteration in HDF_septal–lateral_ nor in HDF_inferior–anterior_ was observed at the E‐wave and at the A‐wave peaks. As a result, in systole, the HDF vector remained aligned with LVOT despite the depressed magnitude; in diastole, it was not aligned with the LV long‐axis (Fig. 7c). These quantitative results were confirmed by the intergroup statistical analysis of HDF orientation, as quantified by the *R*_RMS_ ratio (Fig. 8, Table S3 in the Supplemental Material): *R*_RMS_ was significantly higher in ICM patients vs. controls both in systole and in diastole, confirming that in ICM the HDF was less aligned with the main basal–apical orientation of the diastolic flow.

In AL‐CA patients, the time‐course of the group‐averaged HDF components had minor differences vs. control subjects (Fig. 6c): HDF_basal–apical_ and HDF_septal–lateral_ were slightly reduced at peak systole, while the corresponding E‐wave and A‐wave peaks were slightly amplified and the HDF_basal–apical_ E‐wave had a delayed onset and a significantly shorter duration. The intergroup statistical analysis confirmed the absence of statistically meaningful differences in any HDF component at peak systole (*P* = 0.18, 0.06, and 0.28 for HDF_basal–apical_, HDF_septal–lateral_, and HDF_inferior‐anterior_, respectively) or at the E‐wave (*P* = 0.11, 0.74, and 0.99 for HDF_basal–apical_, HDF_septal–lateral_, and HDF_inferior–anterior_, respectively) and A‐wave (*P* = 0.99 and 0.81 for HDF_basal–apical_ and HDF_septal–lateral_, respectively) diastolic peaks (Table S2 in the Supplemental Material). As a result, the HDF vector remained aligned as in controls in both phases of the cardiac cycle (Fig. 7d).

The *R*_RMS_ ratio revealed a significant increase vs. controls only in systole, and it was preserved in diastole (Fig. 8, Table S3 in the Supplemental Material).

### Interoperator Variability

Over the 15 selected subjects and over the whole cardiac cycle, the comparison between the binary masks obtained by the two independent operators yielded a DSC with a medial value of 0.90 and an interquartile range of 0.87–0.93 (Table S4 in the Supplemental Material). Also, strong linear correlation was reported for KE_V_ and HDF (*r* ^2^ ≥ 0.92), with the strongest level of agreement (*r* ^2^ = 0.99) in diastole (Fig. S4 in the Supplemental Material). Further details are available in Appendix S1 in the Supplemental Material.

## Discussion

The subdivision of LV flow components,^5^ intracavitary blood energetics,^8^, ^22^ and the pressure‐driven HDF^9^ represent three complementary aspects of LV fluid dynamics, potentially related to LV wall function. The present 4D flow study sought to combine these flow‐specific measures in a comprehensive analysis, testing its usefulness in exploring the complex interplay between intracavitary flow and wall function in two pathologies with different patterns of LV remodeling, such as ICM and AL‐CA.

### Preliminary Insight into ICM Physiopathology

In general, ICM is characterized by morphological LV alterations localized at the site of ischemic injury but also potentially affecting the remote myocardium. As a result, LV pump function is progressively impaired, as commonly quantified through global indices (eg, SV, EF, and global longitudinal strain [GLS]).^31^

Our ICM patients suffered from anterior myocardial infarction; as such, they were characterized by an extended scar region.^32^ Consistently, their systolic function was significantly impaired, as evident from a marked reduction in systolic global KE_V_ vs. healthy controls and consistently with previous studies.^12^, ^13^ KE_V_ regional analysis showed a significant reduction in the systolic KE_V_ peaks in the mid and basal regions vs. controls. The former was more severely affected, consistently with the location of the original ischemic injury, but the latter was still notably reduced, highlighting the impact of post‐infarct injury on the remote, although not scarred, myocardium.^33^ In each LV region, the reduction in KE_V_ was paired by a proportional reduction in EL_V_, suggesting that the systolic intracavitary blood flow is characterized by lower velocities, but with no evident changes in the velocity field pattern (e.g., vortical structures) that would lead to increased viscous dissipation. Of note, the reliability of regional data was allowed for by our patient‐specific subdivision of the LV chamber, which proved effective in correctly distinguishing the apical, mid‐cavity, and basal LV regions, despite the heterogeneous LV remodeling patterns affecting our ICM patients.^33^

Moreover, the SV composition was different compared to healthy controls due to the derangement of flow component redistribution: there was a reduction in the DF component, which was recently found by Stoll et al to be comparable between ICM and apparently compensated dilated cardiomyopathy patients.^19^ Of note, in our ICM cohort, the decrease in DF and DE was also paralleled by the increase in RV and RI flow components, whereas in Stoll *et al*.'s cohort^19^ the decrease in DF corresponded to a similar increase in the RV component only. This difference could depend on both extension and localization of ICM injury.

Analysis of HDF can yield the link between derangements in intracavitary systolic blood flow and the depressed LV function in ICM patients.^30^ At peak systole, HDF_basal–apical_ was almost absent and reduced on average by approximately one half vs. controls. This likely reflects a severely reduced “piston‐like” motion of the valvular plane toward the apex and thus the inability to effectively move blood from the sub‐mitral region toward the apex at the onset of systole, and from the more apical regions toward the outflow tract at peak systole.^13^ Concomitantly, HDF_inferior–anterior_ was barely present, reflecting the reduction in anterior wall contractility, and there was a milder reduction of HDF_septal–lateral_ leading to abnormally high *R*_RMS,systole_ values.

Altogether, the different fluid dynamic parameters stress that ICM is a systolic dysfunction, yet derangements in the diastolic phase are also present.

At diastole, LV energetics showed that the KE_V_ peak in the mid‐cavity region associated with the E‐wave was delayed with respect to the corresponding peak in the basal region, whereas no significant delay was observed in controls. This is consistent with the increased duration of the E‐wave peak that was observed in the flow rate time‐course compared to controls. Also, it is consistent with the observations by Garg *et al*.,^13^ who suggested that it is linked with a delayed E‐wave propagation from basal to mid LV regions in ICM patients, leading to a delayed filling of the LV. This anomaly is paralleled by the almost complete disappearance of the two positive peaks in HDF_basal–apical_ that were synchronous to the E‐wave and the A‐wave in healthy controls. As a result, the value of *R*_RMS,diastole_ was almost doubled compared to healthy controls. This lack of transfer of momentum in the basal–apical direction is consistent with the presence of blood RV that remains confined in the dilated and akinetic mid‐apical regions.

### Preliminary Insight into AL‐CA Physiopathology

In our AL‐CA cohort, compared to healthy controls, we observed derangements in LV diastolic fluid dynamics. Specifically, the energetics analysis showed a significant reduction in the E‐wave peak of global and basal KE_V_. Also, while in healthy controls, a valley clearly separated the E‐wave and A‐wave peaks in KE_V_ time‐course, in AL‐CA patients this valley almost disappeared, suggesting the preservation of the time‐averaged value of KE_V_ over the diastolic phase. This is consistent with the computed time‐course of the diastolic flow rate, which in AL‐CA was characterized by a markedly reduced E‐wave peak and absence of a clear distinction between the E‐wave and A‐wave peaks. This behavior is consistent with the reduced volume entering the LV in diastole and hence significantly reduced SV in AL‐CA patients.

The derangements in diastolic LV energetics were not paralleled by anomalies in flow subdivision, thus suggesting that flow organization might not be a suitable marker to highlight AL‐CA‐related fluid dynamic derangements. On the contrary, a more subtle feature was noticed during LV relaxation in HDF_basal–apical_ over the end of systole and the onset of diastole. In controls, we obtained two negative peaks separated by a short notch at the transition between systole and diastole; the earlier peak was characterized by a higher amplitude and was concomitant to the closure of the aortic valve, and the later peak had a smaller amplitude and anticipated the onset of the E‐wave. This pattern is consistent with the observations by Arvidsson et al in healthy volunteers and elite athletes.^14^ In AL‐CA patients, the two negative peaks were still present, but the pattern was altered: the time extent of the two peaks was dilated; the earlier peak was reduced in magnitude compared to healthy controls and anticipated before aortic valve closure, and the second peak was delayed as the notch between the two peaks was not as instantaneous as in the healthy volunteers.

To pinpoint HDF_basal–apical_ alterations at the onset of diastole in AL‐CA patients, as proposed by Arvidsson *et al*.,^14^ we quantified the contribution of HDF_basal–apical_ to early diastolic chamber filling by integrating the time‐course of this HDF component from aortic valve closure, identified as the time‐point with zero flow rate at end‐systole, to the time‐point with zero HDF_basal–apical_ (Fig. S5 in the Supplemental Material). This early diastolic filling impulse, normalized to LV chamber volume, proved to be nearly two thirds higher in AL‐CA patients than in controls with values equal to 4.58 × 10^−2^ and 2.77 × 10^−2^ Ns/L, respectively. This mismatch, given the statistically negligible differences of intracavitary volume between controls and AL‐CA patients, may reflect the reduced diastolic LV compliance during early filling in AL‐CA patients. If this outcome was confirmed by the analysis of larger cohorts of subjects, this index may be considered attractive as it is quantitative and synthetic, and it can be computed in a simple and repeatable way. The observed diastolic derangements in both KE_V_ and HDFs may be signs of deteriorating diastolic function in AL‐CA,^34^ despite no evident anomalies in LV flow subdivision.

Moreover, a significant reduction in diastolic EL_V_, although localized in the basal region, was found with respect to healthy controls. This reduction should not be interpreted as an energetically more efficient filling in AL‐CA patients; in fact, this parameter reflects the phenomena occurring in the bulk of the blood flow and does not account for the dissipative phenomena occurring in the boundary layer at the endocardial level, largely contributing to the actual amount of dissipated energy.^35^ Instead, the decrease in EL_V_ may be due to the absence or reduction of physiological vortical structures in the bulk flow during diastolic filling, potentially due to the reduced compliance of the LV. Of note, the decrease in EL_V_ was mainly localized in the basal region, possibly because of an absent or mitigated toroidal vortical structure that characterizes the sub‐mitral region of healthy subjects during LV filling.^36^

Even if AL‐CA is mostly associated with diastolic dysfunction, slight fluid dynamic derangements were also detected in systole. Global KE_V_ remained comparable to controls, but with an anomalous regional repartition: KE_V_ median value was reduced in the basal region and increased in the mid region. Concomitantly, *R*_RMS_ significantly increased. This result may suggest that:

1. In AL‐CA patients, the LV wall has a slightly impaired capability of transferring momentum to the intracavitary blood rapidly, which is reflected in a reduced net motion of blood toward the LVOT even if the overall amount of KE is preserved or even increased. This interpretation is consistent with the reduced flow rate recorded in these patients, and with the depressed SV reported^37^ for AL‐CA patients. Also, the increase in *R*_RMS_ is due to a decrease in the RMS of HDF_basal–apical_, which is consistent with the reduced GLS observed by Buss *et al*.^38^
2. The impairment may be mostly associated with the basal region, and the LV wall tries to compensate for it by an extra pumping action of its mid portion. This speculation may be consistent with previous MRI findings in cardiac amyloidosis^39^: tissue changes detected by LGE often show a base‐to‐apex gradient possibly impacting longitudinal fibers contractility; this can be paralleled by a preserved or supranormal longitudinal strain in the apical segments, leading to the so‐called apical sparing.^40^

### Potential Value of a Comprehensive Fluid Dynamic Analysis

The selected fluid dynamic parameters have complementary features and can elucidate different aspects of LV function:

1. KE and EL provide volume‐averaged global (over the LV chamber) or regional (base, mid‐cavity, and apex) information on the LV systolic function and on the presence of velocity patterns with relevant gradients, such as vortical structures.^24^ However, they yield no information about the interplay between consecutive phases. Also, because they are scalar and nondirectional quantities, their alterations cannot be directly related to regional LV dysfunction, even when computed at the base, mid, and apical LV regions^13^: for example, the akinesia of any segment of the mid LV could cause a decrease in systolic mid‐ventricular KE.
2. HDF, which is a 3D vector providing information on the direction of the transfer of momentum from LV wall to cavity,^14^ may be exploited to identify the segment that is not inducing blood acceleration (in systole) or that is not absorbing momentum (in diastole) owing to impaired compliance. Yet, HDF provides a global information over the LV chamber.
3. Flow component analysis allows for exploring the interplay between consecutive cardiac cycles.

The analysis of only one of these parameters could be insufficient to highlight LV function derangements: for instance, based on flow component analysis only, LV function in AL‐CA patients would not be detected as significantly altered. Even when not insufficient, it still is not as powerful as the combined analysis of multiple parameters: in our ICM patients, the analysis of HDF components and of KE_V_ together provided an exhaustive understanding of the post‐ischemic scenario; HDF highlighted the anterior location of the infarcted region, which could not be done by the analysis of KE_V_, while the regional analysis of KE_V_ allowed to assess the impact on scarred myocardium as well as on remote myocardium, which could not be done based on HDF quantification. Likewise, for our AL‐CA patients, only the joined analysis of the peaks in regional KE_V_ and of *R*_RMS_ allowed us for a reasonable interpretation of the mechanism underlying the impairment of systolic LV function in a pathology typically used as an example of diastolic dysfunction.

Future work is required to explore if the proposed indexes could be useful to monitor disease progression, as well as the effects of therapy during follow‐up. It also remains to be tested whether their alteration could allow for an earlier detection of changes in LV function compared to standard functional indexes, at least in the two pathological scenarios herein considered. The seminal study by Pedrizzetti *et al*.,^36^ despite being focused on different populations, suggests that this might be the chance.

### Limitations

The study population was relatively small; therefore, our results are preliminary. Also, both groups of patients were heterogeneous: ICM patients, although all affected by the anterior infarction of comparable severity, were heterogeneous in terms of LV characteristics and remodeling severity, and AL‐CA patients had different levels of cardiac involvement. However, despite the limited sample size, this study shows the potential of 4D flow in providing a noninvasive, time‐efficient and multifaceted quantification of specific hemodynamic parameters, whose alterations can be associated with different LV remodeling patterns. Despite their intragroup variability, our ICM and AL‐CA cohorts represent not only two clinically relevant scenarios, but also two opposite LV remodeling mechanisms.

In addition, the limited spatial and temporal resolution of 4D flow acquisitions may impact the granularity of the computed parameters. This may inevitably affect the velocity derivatives computation, and thus the accuracy of computed EL^22^ and pressure gradients.^9^ However, the entire population was assessed with the same 4D flow sequence to allow for intergroup comparisons, hence the alterations observed in ICM and AL‐CA patients vs. healthy controls remain valid. Finally, 4D flow was acquired in controls without the use of contrast agent and this might affect the signal‐to‐noise ratio.

## Conclusion

In a preliminary cohort of patients, 4D flow‐based quantification of LV fluid dynamics characterized intracavitary flow derangements associated with ICM and AL‐CA, selected as representative of systolic and diastolic dysfunction, respectively.

In particular, the analysis of alterations in KE_V_ and HDF provided consistent and complementary information on the interplay between intracardiac blood fluid dynamics and LV wall function.

## Supporting information


**Appendix S1** Supplementary methods.
**Table S1** LV peak kinetic energy normalized to LV volume for the study population, evaluated for the global LV, basal LV, and mid LV.
**Table S2** LV peak hemodynamic forces, for the basal–apical, septal–lateral, and inferior–anterior component, normalized to LV volume for the study population.
**Table S3** *R*_RMS_ ratio for the study population.
**Table S4** Reproducibility analysis.
**Fig. S1** Exemplification of long‐axis CMR views tracing of papillary muscles (tip and base), mitral and aortic valves, and apex for the left ventricle at end diastole.
**Fig. S2** Schematic of the four flow components of the left ventricular blood flow.
**Fig. S3** Definition of hemodynamic force directions.
**Fig. S4** Peak systolic and peak diastolic values of KE_V_ **(a, b)** and HDF_V_ components **(c–h)** extracted from the segmentation performed by two operators.
**Fig. S5** Quantification of the early diastolic filling impulse for *I*_BA_.
**Fig. S6** Parameters required to compute the roto‐translation matrix **M**.

**Video S1** Left ventricular flow component visualization for one healthy control, one ischemic cardiomyopathy patient, and one cardiac amyloidosis patient. Direct flow is indicated in green, retained inflow in yellow, delayed ejection flow in blue, and residual volume in red.
