# Supplementary material for: Comparison of Four‐Dimensional Magnetic Resonance Imaging Analysis of Left Ventricular Fluid Dynamics and Energetics in Ischemic and Restrictive Cardiomyopathies
Source: J Magn Reson Imaging. 2022 Jan 24;56(4):1157–70. doi: 10.1002/jmri.28076 (PMC9541919; doi:10.1002/jmri.28076)
Supplement: Supplementary file 1 — Appendix S1 Supplementary methods. Table S1 LV peak kinetic energy normalized to LV volume for the study population, evaluated for the global LV, basal LV, and mid LV. Table S2 LV peak hemodynamic forces, for the basal–apical, septal–lateral, and inferior–anterior component, normalized to LV volume for the study population. Table S3 R RMS ratio for the study population. Table S4 Reproducibility analysis. Fig. S1 Exemplification of long‐axis CMR views tracing of papillary muscles (tip and base), mitral and aortic valves, and apex for the left ventricle at end diastole. Fig. S2 Schematic of the four flow components of the left ventricular blood flow. Fig. S3 Definition of hemodynamic force directions. Fig. S4 Peak systolic and peak diastolic values of KEV (a, b) and HDFV components (c–h) extracted from the segmentation performed by two operators. Fig. S5 Quantification of the early diastolic filling impulse for I BA. Fig. S6 Parameters required to compute the roto‐translation matrix M. [file JMRI-56-1157-s002.docx]

Online supplement to

**Comparison of four-dimensional magnetic resonance imaging analysis of left ventricular fluid dynamics and energetics in ischemic and restrictive cardiomyopathiesSupplementary Methods**

**Mathematical methods
*Energetic variables computation from 4D flow***

Blood kinetic energy (KE), i.e., the energy that a volume of blood possesses due to its motion, is computed at each time frame as:

|  | $KE=\sum_{i=1}^{N} \frac{1}{2}\rho V_{i}v_{i}^{2}$ | (**S1**) |
| --- | --- | --- |

where $\rho$ is the blood density (equal to 1025 kg/m^3^), $V_{i}$ the $i^{th}$ voxel volume, $v$ the velocity magnitude and $N$ the total number of voxels within the region of interest (ROI).

Viscous energy loss ($EL_{T}$), the blood KE irreversibly lost due to frictional viscous forces over a period T (i.e., systole, diastole), was computed as:

|  | $EL_{T}=\int_{t_{0}}^{\bar{t}} \dot{EL}dt$ | (**S2**) |
| --- | --- | --- |

The rate of viscous energy loss ($\dot{EL}$), evaluated in mW for the time frame *t*, can be computed as:

|  | $\dot{EL_{t}}=\mu_{Quemada}\sum_{i=1}^{N} \phi_{v}V_{i}$ | (**S3**) |
| --- | --- | --- |

where $\mu_{Quemada}$ is blood viscosity obtained through the Quemada model (1) (see **Non-Newtonian viscosity model**). The viscous dissipation function $\phi_{v}$ quantifies the rate of viscosity-induced energy dissipation per unit volume (2) and is computed from the Navier-Stokes energy balance equation as:

| $\phi_{v}=2\left[ \left( \frac{\partial u}{\partial x} \right)^{2}+\left( \frac{\partial v}{\partial y} \right)^{2}+\left( \frac{\partial w}{\partial z} \right)^{2}-\frac{1}{3}\left( \nabla\cdot\mathbf{v} \right)^{2} \right]+\left( \frac{\partial v}{\partial x}+\frac{\partial u}{\partial y} \right)^{2}+\left( \frac{\partial w}{\partial y}+\frac{\partial v}{\partial z} \right)^{2}+\left( \frac{\partial u}{\partial z}+\frac{\partial w}{\partial x} \right)^{2}$ | (**S4**) |
| --- | --- |

where $\nabla\cdot\mathbf{v}$ is the divergence of the velocity field and thus equal to:

|  | $\nabla\cdot\mathbf{v}=\frac{\partial u}{\partial x}+\frac{\partial v}{\partial y}+\frac{\partial w}{\partial z}$ | (**S5**) |
| --- | --- | --- |

***Non-Newtonian viscosity model***

The Quemada non-Newtonian model (3) considers the shear thinning behavior of blood and relates blood viscosity to hematocrit (Hct) and $\dot{\gamma}$ through the following formula:

|  | $\mu_{Quemada}=\frac{\mu_{plasma}}{\left( 1-\frac{Hct\cdot k_{eq}}{2} \right)^{2}}$ | (**S6**) |
| --- | --- | --- |

where $\mu_{plasma}$ is the plasma viscosity and $k_{eq}$ is the intrinsic viscosity of erythrocytes (4), evaluated as:

|  | $k_{eq}=\frac{k_{0}+k_{\infty}\cdot\sqrt{\dot{\gamma}/\dot{\gamma}_{c}}}{1+\sqrt{\dot{\gamma}/\dot{\gamma}_{c}}}$ | (**S7**) |
| --- | --- | --- |

where $k_{0}$ and $k_{\infty}$ are the maximum volume fraction at zero and infinite $\dot{\gamma}$, and $\dot{\gamma_{c}}$ is the critical shear rate for erythrocytes agglomeration (5). Despite being potentially influenced by hematocrit (6), the values of $\mu_{plasma}$, $\dot{\gamma_{c}}, k_{0}$ and $k_{\infty}$ were kept constant ($\mu_{plasma}=1.28 cP$, $\dot{\gamma_{c}}=4.2 s^{-1}$, $k_{0}=4.01$ and $k_{\infty}=1.77$) (4), where these values were obtained from blood samples with minor hematocrit variability (44 ± 1%) and with no cardioactive medication within a week prior to the test. In non-Newtonian models, $\dot{\gamma}$ is defined as a function of the second invariant ($D_{II}$) of the rate of deformation tensor (**D**) (7):

|  | $\dot{\gamma}=2\sqrt{D_{II}}$ | (**S8**) |
| --- | --- | --- |

The rate of deformation tensor is the symmetric part of the velocity gradient tensor $\mathbf{L}$, which in Cartesian coordinates is given by:

|  | $\mathbf{L}=\left[ \begin{matrix} \frac{\partial u}{\partial x} & \frac{\partial u}{\partial y} & \frac{\partial u}{\partial z} \\ \frac{\partial v}{\partial x} & \frac{\partial v}{\partial y} & \frac{\partial v}{\partial z} \\ \frac{\partial w}{\partial x} & \frac{\partial w}{\partial y} & \frac{\partial w}{\partial z} \end{matrix} \right]$ | (**S9**) |
| --- | --- | --- |

where $u$, $v$, and $w$ are velocities in the $x$, $y$, and $z$ direction, respectively (8). $\mathbf{D}$ is hence computed as:

|  | $\mathbf{D}=\frac{1}{2}\left( \mathbf{L}+\mathbf{L}^{T} \right)=\left[ \begin{matrix} \frac{\partial u}{dx} & \frac{1}{2}\left( \frac{\partial u}{\partial y}+\frac{\partial v}{\partial x} \right) & \frac{1}{2}\left( \frac{\partial u}{\partial z}+\frac{\partial w}{\partial x} \right) \\ \frac{1}{2}\left( \frac{\partial u}{\partial y}+\frac{\partial v}{\partial x} \right) & \frac{\partial v}{\partial y} & \frac{1}{2}\left( \frac{\partial v}{\partial z}+\frac{\partial w}{\partial y} \right) \\ \frac{1}{2}\left( \frac{\partial u}{\partial z}+\frac{\partial w}{\partial x} \right) & \frac{1}{2}\left( \frac{\partial v}{\partial z}+\frac{\partial w}{\partial y} \right) & \frac{\partial w}{\partial z} \end{matrix} \right]$ | (**S10**) |
| --- | --- | --- |

And $D_{II}$ is obtained as:

|  | $D_{II}=\frac{1}{2}\left[ \left( tr\left( \mathbf{D} \right) \right)^{2}-tr\left( \mathbf{D}^{2} \right) \right]=\frac{1}{2}\left[ \left( \frac{\partial u}{\partial x}+\frac{\partial v}{\partial y}+\frac{\partial w}{\partial z} \right)^{2}-\mathbf{D}:\mathbf{D}^{T} \right]$ | (**S11**) |
| --- | --- | --- |

which, for a three-dimensional geometry, is equal to:

| $D_{II}=\left( \frac{\partial u}{\partial x}\frac{\partial v}{\partial y}+\frac{\partial v}{\partial y}\frac{\partial w}{\partial z}+\frac{\partial u}{\partial x}\frac{\partial w}{\partial z} \right)-\frac{1}{4}\left( \frac{\partial u}{\partial y}+\frac{\partial v}{\partial x} \right)^{2}-{\frac{1}{4}\left( \frac{\partial u}{\partial z}+\frac{\partial w}{\partial x} \right)}^{2}-\frac{1}{4}\left( \frac{\partial v}{\partial z}+\frac{\partial w}{\partial y} \right)^{2}$ | (**S12**) |
| --- | --- |

***Hemodynamic force computation***

The pressure gradient ($\boldsymbol{b}$) was computed from the Navier-Stokes equation, according to (9), as:

|  | $\boldsymbol{b}=-\rho\frac{\partial\boldsymbol{v}}{\partial t}-\rho\left( \boldsymbol{v}\cdot\nabla\boldsymbol{v} \right)+\mu\nabla^{2}\boldsymbol{v}$ | (**S13**) |
| --- | --- | --- |

First order derivatives were approximated by 4^th^ order schemes in the internal points of the domain, while 3^rd^ order schemes were used on the boundaries. Similarly, second order derivatives were approximated using 4^th^ order and 3^rd^ order schemes were applicable and 2^nd^ order schemes on the boundaries. Time derivatives were calculated using 2^nd^ order schemes.

The hemodynamic force was computed as the integral of $\boldsymbol{b}$ over the left ventricle and thus computed in [N]. The directions onto which project the hemodynamic force vector are defined according to (10) and as depicted in **Figure S3**.

***R_RMS_ ratio***

The temporal root mean square (RMS) of the hemodynamic force was evaluated as:

|  | $RMS=\sqrt{\frac{1}{N}\sum_{t=1}^{N} \left\vert{HDF}_{t} \right\vert^{2}}$ | (**S14**) |
| --- | --- | --- |

where $N$ corresponds to the number of time frames in a cardiac phase, $HDF$ is the force in the time frame $t$. The relative magnitude of transverse (inferior-anterior and septal-lateral) and longitudinal (basal-apical) forces was quantified as the ratio between transverse and longitudinal forces:

|  | $R_{RMS}=\frac{\sqrt{RMS_{inferior-anterior}^{2}+RMS_{septal-lateral}^{2}}}{RMS_{basal-apical}}=\frac{RMS_{transversal}}{RMS_{basal-apical}}$ | (**S15**) |
| --- | --- | --- |

where $RMS_{basal-apical}$, $RMS_{inferior-anterior}$ and $RMS_{septal-lateral}$ are the RMS of the force components in the basal-apical, inferior-anterior and septal-lateral directions, respectively.

**Spatial and temporal co-registration**

Spatial co-registration is performed through a roto-translation matrix ***M***. This matrix is built from the DICOM file parameters (**Figure S6**):

- *Image Position Patient (ipp)*: $x$, $y$, $z$ coordinates of the centre of the first voxel, expressed in [mm] with respect to the global reference system;
- *Image Orientation Patient (iop)*: coordinates of the two unit vectors defining the image plane, thus the direction cosines of rows and columns. It is a vector of six elements: the first three concern the direction of the first row of the grid (*d_1_*), while the remaining three concern the direction of the first column (*d_2_*). The third direction (*d_3_*) is obtained from the vector product of the previously defined unit vectors *d_1_* and *d_2_*. These three unitary vectors define a orthonormal frame centred in the origin of the image (identified by *ipp*);
- *Pixel Spacing (ps)*: distance between the centres of adjacent voxels, expressed in [mm];
- *Slice Thickness (st)*: nominal thickness of the 4D Flow slice, expressed in [mm].

***M*** is thus given by the product of three matrixes:

- rotation matrix (***R***)

|  | $d_{1}=iop\left( 1:3 \right)$ $d_{2}=iop\left( 4:6 \right)$ $d_{3}=d_{1}\times d_{2}$ | (**S16**) |
| --- | --- | --- |
|  | $\boldsymbol{R}=\left[ \begin{matrix} \begin{matrix} d_{1}(1) & d_{2}(1) \\ d_{1}(2) & d_{2}(2) \end{matrix} & \begin{matrix} d_{3}(1) & 0 \\ d_{3}(2) & 0 \end{matrix} \\ \begin{matrix} d_{1}(3) & d_{2}(3) \\ 0 & 0 \end{matrix} & \begin{matrix} d_{3}(3) & 0 \\ 0 & 1 \end{matrix} \end{matrix} \right]$ | (**S17**) |

- translation matrix (***T***):

|  | $\boldsymbol{T}=\left[ \begin{matrix} \begin{matrix} 1 & 0 \\ 0 & 1 \end{matrix} & \begin{matrix} 0 & ipp(1) \\ 0 & ipp(2) \end{matrix} \\ \begin{matrix} 0 & 0 \\ 0 & 0 \end{matrix} & \begin{matrix} 1 & ipp(3) \\ 0 & 1 \end{matrix} \end{matrix} \right]$ | (**S17**) |
| --- | --- | --- |

- scaling matrix (***S***):

|  | $\boldsymbol{S}=\left[ \begin{matrix} \begin{matrix} ps\left( 1 \right) & 0 \\ 0 & ps\left( 2 \right) \end{matrix} & \begin{matrix} 0 & 0 \\ 0 & 0 \end{matrix} \\ \begin{matrix} 0 & 0 \\ 0 & 0 \end{matrix} & \begin{matrix} st & 0 \\ 0 & 1 \end{matrix} \end{matrix} \right]$ | (**S18**) |
| --- | --- | --- |
|  | $\boldsymbol{M=R\cdot T\cdot S}$ | (**S19**) |

***M*** allows transforming the local coordinates obtained from the LV segmentation to the global coordinates, defined with respect to the reference system of the MR.

Temporal co-registration was performed only in the case of different number of reconstructed phases between cine and 4D Flow acquisitions and exploits the definition of the end systolic (ES_cine_) time frame in the cine stack of images (chosen by the medical doctor). Given the acquired cardiac phases and the length of the cardiac cycle, the temporal resolution for the cine and 4D Flow acquisition is given by:

|  | $temporal resolution \left[ ms \right]=\frac{T_{cycle}}{number of temporal phases}$ | (**S20**) |
| --- | --- | --- |

The end systolic time frame for the 4D Flow acquisition (ES_4DFlow_) is thus defined as the one reporting the lowest temporal difference with respect to ES_cine_. The time delay is computed as:

|  | $\Delta t=t\left( ES_{cine} \right)-t\left( ES_{4DFlow} \right)$ | (**S21**) |
| --- | --- | --- |

According to the time delay and to the different temporal resolution, the two datasets are synchronized so to obtain the binary LV mask, associated to each 4D Flow phase, being the closest in time.

Any misalignment of the LV ROI, due to patient motion between the cine and the 4D Flow acquisition, was manually corrected for.

**Percentage difference computation**

Percentages differences in terms of hemodynamic variables are computed as:

|  | $\frac{\bar{variable}_{p}^{peak phase}-\bar{variable}_{c}^{peak phase}}{\bar{variable}_{c}^{peak phase}}\cdot100$ | (**S21**) |
| --- | --- | --- |

where *p* stands for pathologic (ICM or AL-CA) and *c* stands for controls.

**Reproducibility analysis**

To evaluate the reproducibility of the LV segmentation through our *in-house* MATLAB tool (Mathworks Inc., Natick, Massachusetts, USA), fifteen subjects were randomly chosen in the study population (5 controls, 5 ICM, 5 AL-CA), and their *cine* CMR images were segmented by two independent operators in a double-blind fashion. Both operators manually segmented the LV endocardial surface at end-diastole; the segmentation was automatically propagated to the subsequent time frames of the sequence, and manual corrections of the automatic procedure were performed when deemed necessary. The LV endocardial contours were automatically registered in space and time with the corresponding 4D Flow data, and at each time frame of the 4D Flow sequence a binary mask was automatically obtained. The LV binary masks obtained by the two operators were quantitatively compared at each time frame by computing the dice score coefficient (DSC) (11), defined as:

|  | $DSC=\frac{2\cdot\left( ROI_{Op1}\cap ROI_{Op2} \right)}{ROI_{Op1}+ROI_{Op2}}$ | (**S22**) |
| --- | --- | --- |

The value of a DSC ranges from 0, indicating no spatial overlap between the two binary masks, to 1, indicating complete overlap (12). For the whole set of 15 subjects and for all the time frames, the median DSC value was equal to 0.90, with an interquartile range of [0.87; 0.93]. The DSC values obtained for each analysed subject over the different time frames are reported in **Table S5** as median [interquartile range].

For the selected population, peak systolic and peak diastolic (i.e., E-wave) values of KE and HDF components were quantified based on the segmentation by the two operators. Linear regression and relative error ratios were used to explore the inter-operator variability. Results are reported in **Figure S4**. Overall, strong linear correlation was found for all the computed parameters (*r^2^* ≥ 0.92). Both KE and HDF reached the highest level of agreement (r^2^ = 0.99) at diastole.

**Duration of the E-wave HDF_Basal-Apical_ peak**

The onset of the E-wave of *HDF_Basal-Apical_* was identified as the local minimum of the curve occurring just after the systolic peak; the subsequent local minimum was recognized as the end of the E-wave course. Accordingly, the duration of the E-wave peak was computed as the time-difference between the onset and the end of the E-wave. The control group reported an E-wave duration of 0.50 ± 0.04 s, while for AL-CA, the duration significantly reduced to 0.43 ± 0.07 s (*p* = 0.016).

**Supplementary Tables**

**Table S1.** LV peak kinetic energy normalized to LV volume for the study population, evaluated for the global LV, basal LV and mid LV. Data expressed as mean ± SD. Unit of normalized kinetic energy: mJ/ml. Two-way ANOVA for repeated measures (Tukey multiple comparisons test): * *p* < 0.05, *vs*. AL-CA; ^§^ *p* < 0.05, *vs*. ICM.

| LV global | ***Peak S*** | ***Peak E*** | ***Peak A*** | *p*_phase_ | *p*_group_ |
| --- | --- | --- | --- | --- | --- |
| **Controls** | 0.041 ± 0.009 ^§^ | 0.037 ± 0.019 ^§,^* | 0.017 ± 0.007 | < 0.0001 | 0.0010 |
| **ICM** | 0.019 ± 0.011 * | 0.014 ± 0.007 | 0.010 ± 0.003 |  |  |
| **AL-CA** | 0.040 ± 0.013 | 0.025 ± 0.013 | 0.019 ± 0.011 |  |  |
| LV basal | ***Peak S*** | ***Peak E*** |  | *p*_phase_ | *p*_group_ |
| **Controls** | 0.062 ± 0.026 ^§^ | 0.057 ± 0.031 ^§,^* | - | < 0.0001 | 0.0099 |
| **ICM** | 0.034 ± 0.018 | 0.021 ± 0.013 | - |  |  |
| **AL-CA** | 0.055 ± 0.018 | 0.029 ± 0.014 | - |  |  |
| LV mid | ***Peak S*** | ***Peak E*** |  | *p*_phase_ | *p*_group_ |
| **Controls** | 0.015 ± 0.009 ^§^ | 0.019 ± 0.009 ^§^ | - | 0.1571 | 0.0014 |
| **ICM** | 0.005 ± 0.002 * | 0.008 ± 0.003 * | - |  |  |
| **AL-CA** | 0.017 ± 0.010 | 0.020 ± 0.013 | - |  |  |

**Table S2.** LV peak hemodynamic forces, for the basal-apical, septal-lateral and inferior-anterior component, normalized to LV volume for the study population. Data expressed as mean ± SD. Unit of normalized hemodynamic force: N/l. Two-way ANOVA for repeated measures (Tukey multiple comparisons test): ^§^ *p* < 0.05, *vs*. ICM, * *p* < 0.05, *vs*. AL-CA.

| *HDF_basal-apical_* [N/l] | **Controls**  (n = 10) | **ICM**  (n = 10) | **AL-CA**  (n = 10) | *p*_phase_ | *p*_group_ |
| --- | --- | --- | --- | --- | --- |
| ***Peak S*** | 3.04 ± 0.83 ^§^ | 1.29 ± 0.49 * | 2.39 ± 0.94 | < 0.0001 | 0.0007 |
| ***Peak E*** | 2.17 ± 0.95 ^§^ | 0.88 ± 0.35 | 1.44 ± 0.73 |  |  |
| ***Peak A*** | 1.65 ± 1.01 | 0.83 ± 0.40 | 1.63 ± 1.10 |  |  |
| *HDF_septal-lateral_* [N/l] | **Controls** | **ICM** | **AL-CA** | *p*_phase_ | *p*_group_ |
| ***Peak S*** | 2.71 ± 1.04 ^§^ | 1.08 ± 0.54 * | 2.17 ± 0.80 | < 0.0001 | 0.0055 |
| ***Peak E*** | 0.39 ± 0.18 | 0.47 ± 0.21 | 0.23 ± 0.12 |  |  |
| ***Peak A*** | 0.34 ± 0.21 | 0.24 ± 0.15 | 0.21 ± 0.13 |  |  |
| *HDF_inferior-anterior_* [N/l] | **Controls** | **ICM** | **AL-CA** | *p*_phase_ | *p*_group_ |
| ***Peak S*** | 1.17 ± 0.54 ^§^ | 0.61 ± 0.40 | 0.87 ± 0.57 | < 0.0001 | 0.2514 |
| ***Peak E*** | 0.39 ± 0.13 | 0.45 ± 0.12 | 0.41 ± 0.24 |  |  |

**Table S3.** $R_{RMS}$ ratio for the study population. Data expressed as mean ± SD. Two-way ANOVA for repeated measures (Tukey multiple comparisons test): ^§^ *p* < 0.05, *vs*. ICM, * *p* < 0.05, *vs*. AL-CA.

|  | **Controls**  (n = 10) | **ICM**  (n = 10) | **AL-CA**  (n = 10) | *p* value  phase | *p* value  group |
| --- | --- | --- | --- | --- | --- |
| $R_{RMS,systole}$ | 0.99 ± 0.22 ^§,^* | 1.33 ± 0.46 | 1.27 ± 0.09 | < 0.0001 | 0.0004 |
| $R_{RMS,diastole}$ | 0.47 ± 0.17 ^§^ | 0.81 ± 0.25 * | 0.41 ± 0.13 |  |  |

**Table S4.** Reproducibility analysis**.** For 15 randomly selected subjects, the time-dependent LV binary masks obtained upon the segmentation of cine-CMR images by two independent and double-blinded operators were compared via dice score coefficient. Data are expressed as median [interquartile range] over the whole cardiac cycle. AL-CA, light-chain cardiac amyloidosis; C, control; ICM, ischemic cardiomyopathy.

| **Dataset** | **Dice score coefficient** |
| --- | --- |
| C_1_ | 0.86 [0.84; 0.87] |
| C_2_ | 0.89 [0.86; 0.91] |
| C_3_ | 0.85 [0.83; 0.87] |
| C_4_ | 0.89 [0.80; 0.90] |
| C_5_ | 0.95 [0.92; 0.96] |
| ICM_1_ | 0.90 [0.89; 0.91] |
| ICM_2_ | 0.93 [0.92; 0.95] |
| ICM_3_ | 0.94 [0.93; 0.95] |
| ICM_4_ | 0.93 [0.92; 0.94] |
| ICM_5_ | 0.90 [0.89; 0.90] |
| AL-CA_1_ | 0.93 [0.93; 0.94] |
| AL-CA_2_ | 0.88 [0.87; 0.89] |
| AL-CA_3_ | 0.89 [0.85; 0.92] |
| AL-CA_4_ | 0.89 [0.88; 0.89] |
| AL-CA_5_ | 0.86 [0.85; 0.87] |

**Supplementary Figures**

**
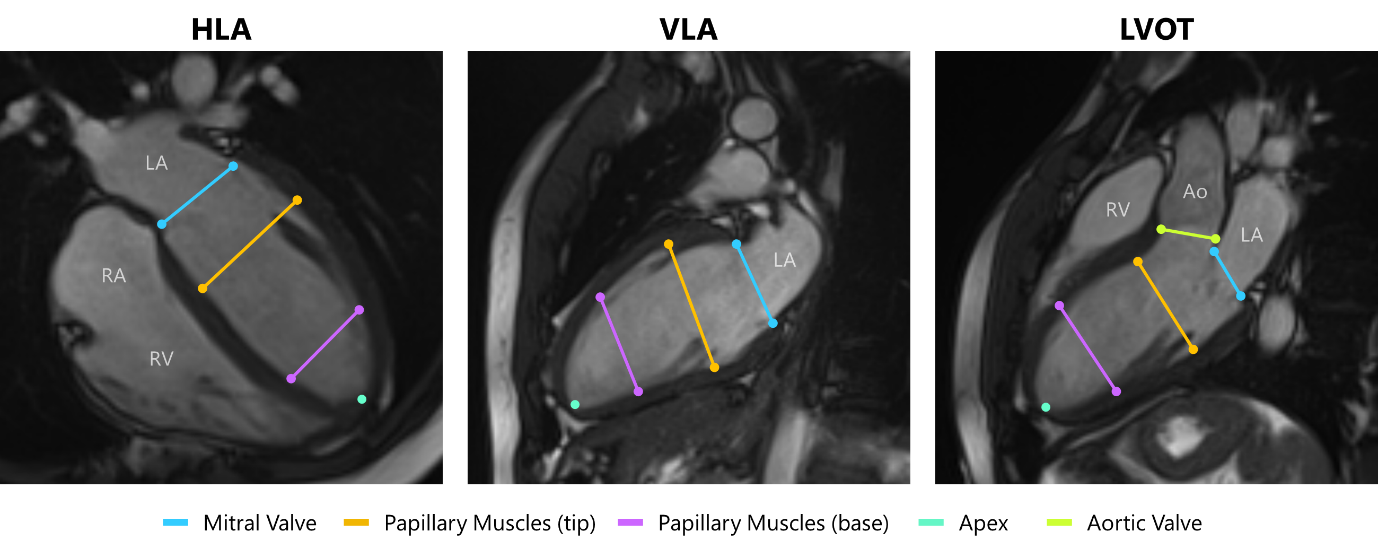
**

**Figure S1.** Exemplification of long-axis CMR views tracing of papillary muscles (tip and base), mitral and aortic valves, and apex for the left ventricle at end diastole. The points in the figure correspond to the ones traced in the software.
Ao, aorta; HLA, horizontal long axis; LA, left atrium; LVOT, left ventricular outflow tract; RA, right atrium; RV, right ventricle; VLA, vertical long axis.

**
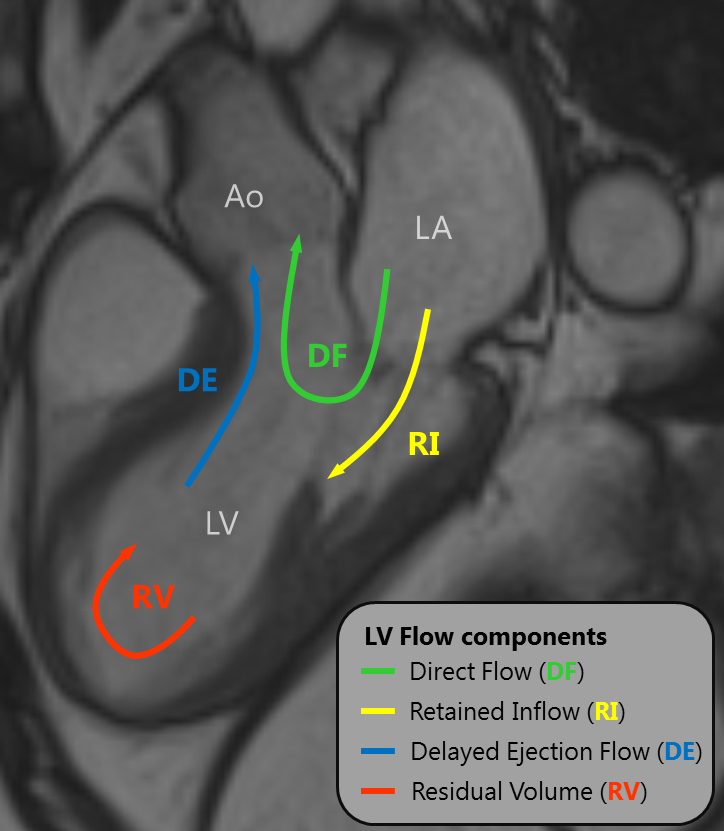
**

**Figure S2.** Schematic of the four flow components of the left ventricular blood flow. Direct Flow (DF, green) is the blood flow that enters the left ventricle during diastole and leaves during systole, for the analysed heartbeat. Retained Inflow (RI, yellow) is the blood that enters the left ventricle during diastole but does not leave during systole in the considered heartbeat. Delayed Ejection Flow (DE, light blue) is the blood that already resides in the left ventricle during diastole and leaves during systole for the analysed heartbeat. Residual Volume (RV, red) is the blood that resides in the LV for at least two cardiac cycles. Ao, aorta; LA, left atrium; LV, left ventricle.


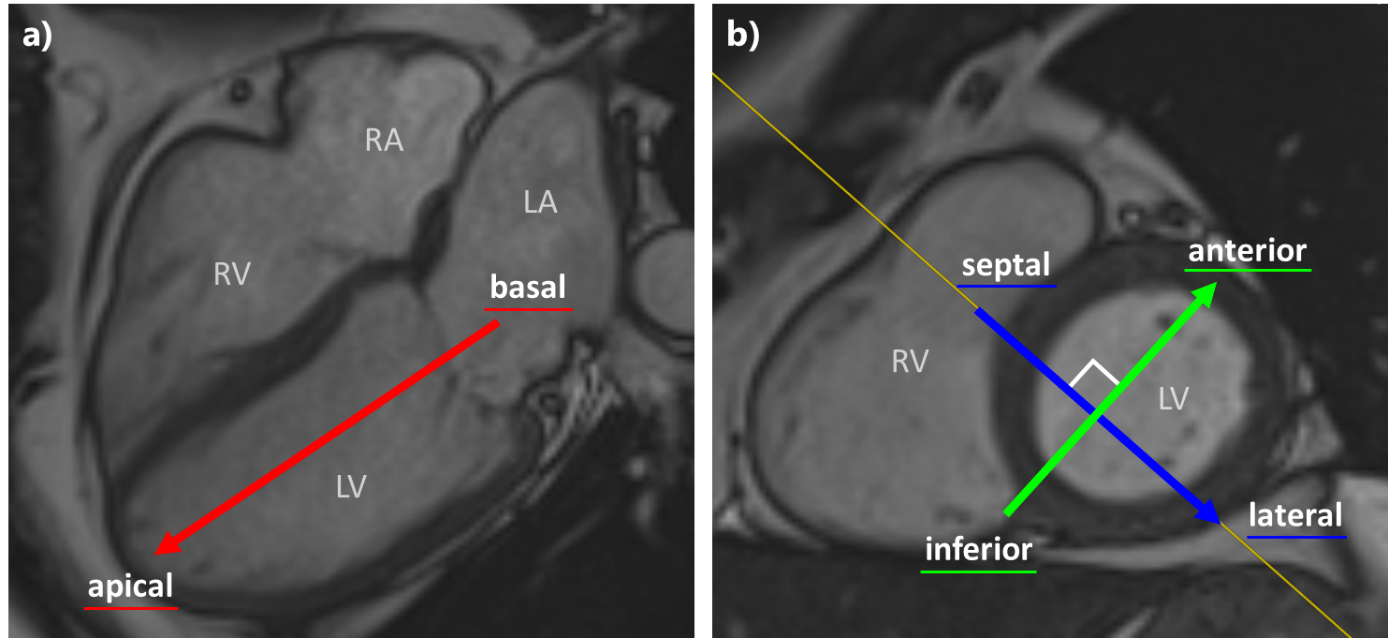


**Figure S3.** Definition of hemodynamic force directions. Panel **a**) shows the basal-apical direction (red). Panel **b**) shows the LV transverse directions: the septal-lateral direction (blue) is aligned with the LVOT slice location (yellow) and the inferior-anterior direction (green) is perpendicular to the previously defined directions (i.e., basal-apical and septal-lateral).
LA, left atrium; LV, left ventricle; RA, right atrium; RV, right ventricle.


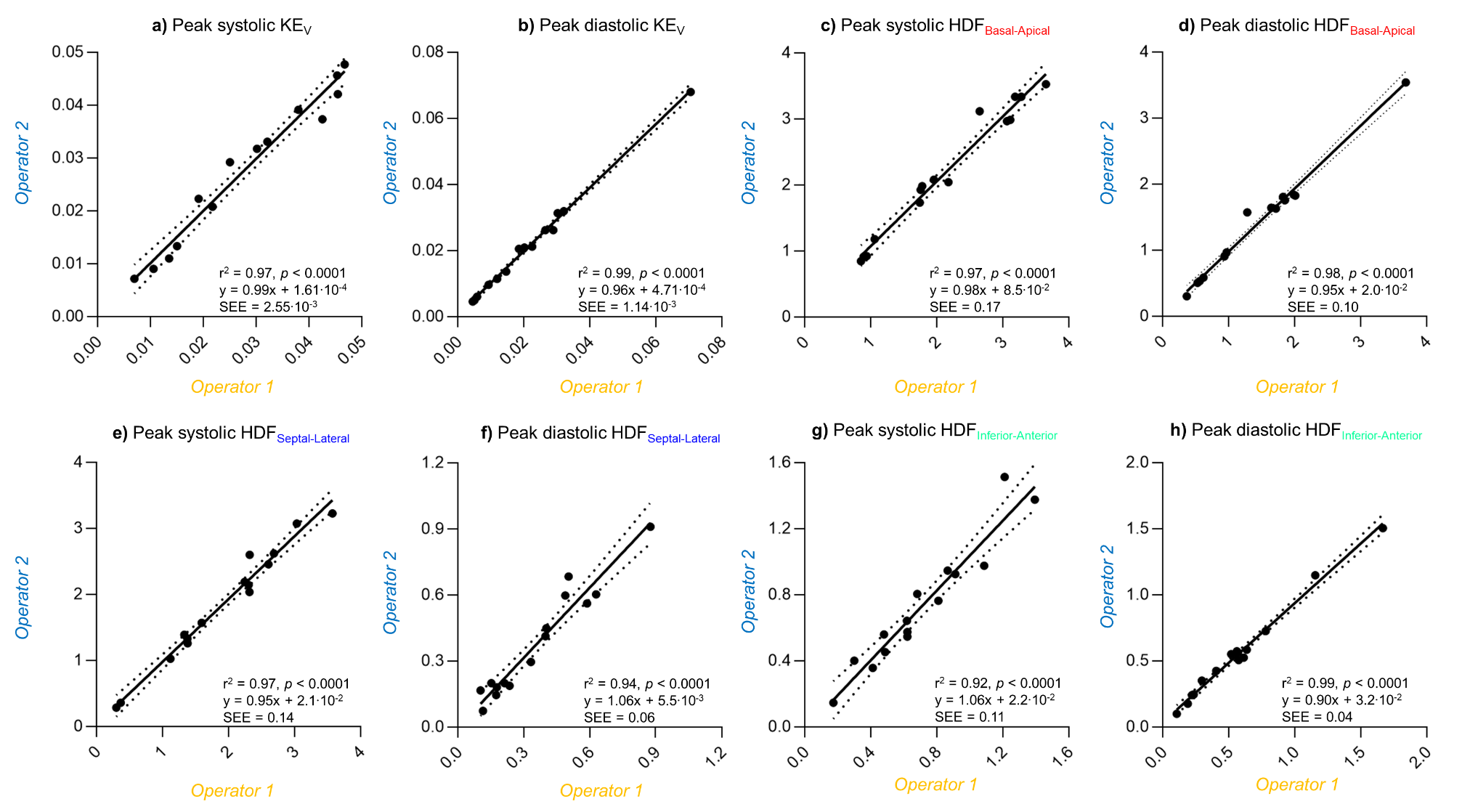


**Figure S4.** Peak systolic and peak diastolic values of KE_V_ (**a, b**) and HDF_V_ components (**c-h**) extracted from the segmentation performed by two operators. Centre continuous line represents the linear regression line with dotted lies delimiting the corresponding 95% confidence band of each best-fit line. KE, kinetic energy; HDF, hemodynamic force; SEE, standard error of estimate.

**
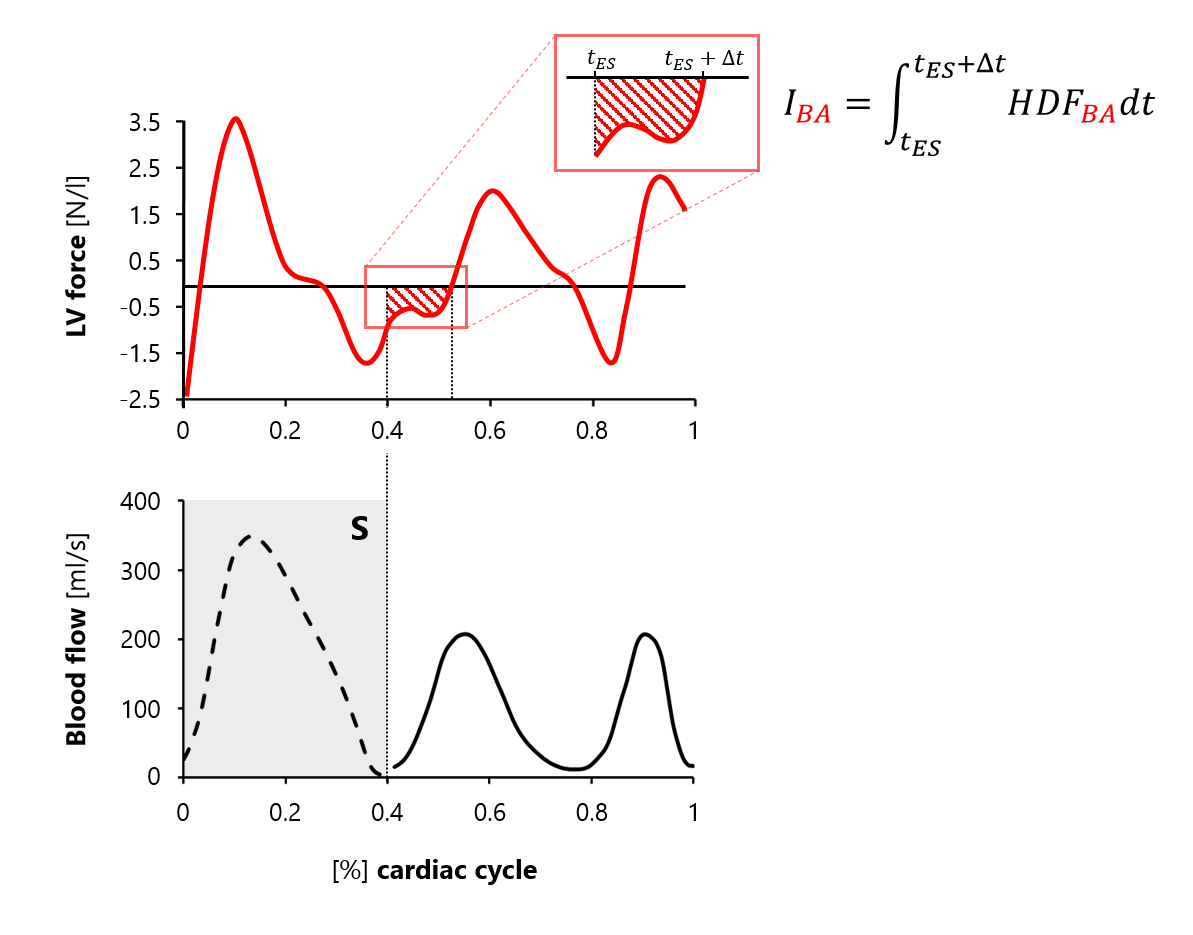
**

**Figure S5.** Quantification of the early diastolic filling impulse for ($I_{BA}$). The hatched area represents the basal-apical impulse, normalized to LV volume (N s/l), exerted on blood after the end systolic time frame (identified from the blood flow curve). The impulse is computed using a trapezoidal numerical integration scheme.
BA, basal-apical; ES, end-systolic time frame; HDF, hemodynamic force; LV, left ventricle; S, systole.


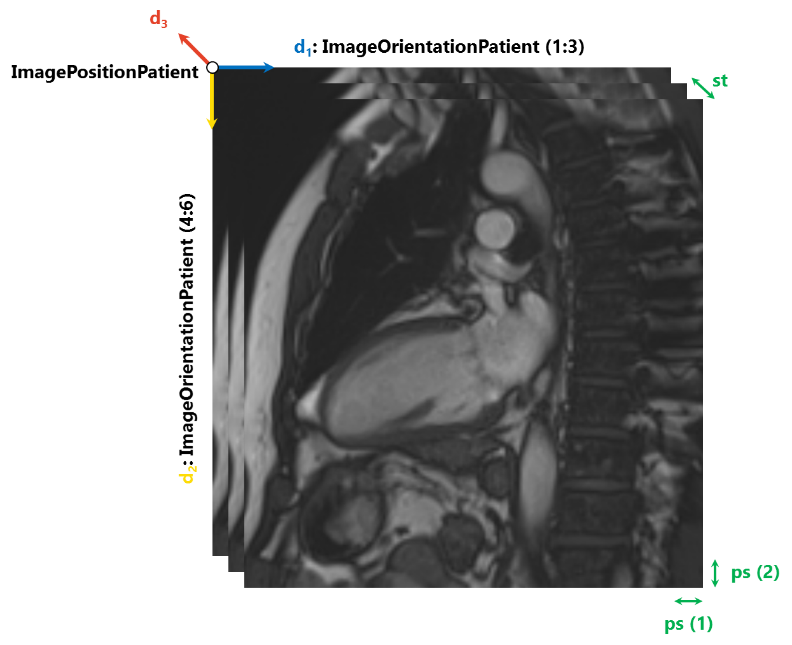


**Figure S6.** Parameters required to compute the roto-translation matrix **M**: *Image Position Patient* (ipp), *Image Orientation Patient* (iop), *Pixel Spacing* (ps) and *Slice Thickness* (st), on a vertical long-axis view of the left ventricle.

**Video S1**. Left ventricular flow component visualisation for one healthy control, one ischemic cardiomyopathy patient and one cardiac amyloidosis patient. Direct flow, green; retained inflow, yellow; delayed ejection flow, blue and residual volume, red.

**References**

1. Riva A, Sturla F, Caimi A, et al.: 4D Flow evaluation of blood non-Newtonian behavior in left ventricle flow analysis. *J Biomech* 2021:110308.

2. Eriksson J, Bolger AF, Ebbers T, Carlhäll CJ: Assessment of left ventricular hemodynamic forces in healthy subjects and patients with dilated cardiomyopathy using 4D flow MRI. *Physiol Rep* 2016; 4:1–12.

3. Quemada D: Laboratoire de Biorhéologie et d ’ Hydrodynamique Physique 1 ), U . E . R . de Physique Université Paris VII Rheology of concentrated disperse systems III . General features of the proposed non-newtonian model . Comparison with experimental data Rheologic. *Rheology* 1978; 653:643–653.

4. Marcinkowska-Gapińska A, Gapinski J, Elikowski W, Jaroszyk F, Kubisz L: Comparison of three rheological models of shear flow behavior studied on blood samples from post-infarction patients. *Med Biol Eng Comput* 2007:837–844.

5. Yeh HH, Barannyk O, Grecov D, Oshkai P: The influence of hematocrit on the hemodynamics of artificial heart valve using fluid-structure interaction analysis. *Comput Biol Med* 2019; 110(April):79–92.

6. Sriram K, Intaglietta M, Tartakovsky DM: Non-Newtonian Flow of Blood in Arterioles: Consequences for Wall Shear Stress Measurements. *Microcirculation* 2014; 21:628–639.

7. Macosko CW: *Rheology Principles, Measurements and Applications*. 1st Editio.; 1994.

8. Hamedi H, Rahimian MH: Numerical Simulation of Non-Newtonian Pseudo-Plastic Fluid in a Micro-Channel Using the Lattice Boltzmann Method. *World J Mech* 2011; 01:231–242.

9. Saitta S, Pirola S, Piatti F, et al.: Evaluation of 4D flow MRI-based non-invasive pressure assessment in aortic coarctations. *J Biomech* 2019; 94:13–21.

10. Arvidsson PM, Töger J, Carlsson M, et al.: Left and right ventricular hemodynamic forces in healthy volunteers and elite athletes assessed with 4D flow magnetic resonance imaging. *Am J Physiol - Hear Circ Physiol* 2017; 312:H314–H328.

11. Tustison N, Gee J: Introducing Dice, Jaccard, and Other Label Overlap Measures To ITK. *Insight J* 2009.

12. Zou KH, Warfield SK, Bharatha A, et al.: Statistical Validation of Image Segmentation Quality Based on a Spatial Overlap Index. *Acad Radiol* 2004; 11:178–189.
